# iDEP: an integrated web application for differential expression and pathway analysis of RNA-Seq data

**DOI:** 10.1186/s12859-018-2486-6

**Published:** 2018-12-19

**Authors:** Steven Xijin Ge, Eun Wo Son, Runan Yao

**Affiliations:** Department of Mathematics and Statistics, South Dakota State University, Box 2225, Brookings, SD 57007 USA

**Keywords:** RNA-seq, Bioinformatics, Web application, Differential gene expression, pathway analysis

## Abstract

**Background:**

RNA-seq is widely used for transcriptomic profiling, but the bioinformatics analysis of resultant data can be time-consuming and challenging, especially for biologists. We aim to streamline the bioinformatic analyses of gene-level data by developing a user-friendly, interactive web application for exploratory data analysis, differential expression, and pathway analysis.

**Results:**

iDEP (integrated Differential Expression and Pathway analysis) seamlessly connects 63 R/Bioconductor packages, 2 web services, and comprehensive annotation and pathway databases for 220 plant and animal species. The workflow can be reproduced by downloading customized R code and related pathway files. As an example, we analyzed an RNA-Seq dataset of lung fibroblasts with Hoxa1 knockdown and revealed the possible roles of SP1 and E2F1 and their target genes, including microRNAs, in blocking G1/S transition. In another example, our analysis shows that in mouse B cells without functional p53, ionizing radiation activates the MYC pathway and its downstream genes involved in cell proliferation, ribosome biogenesis, and non-coding RNA metabolism. In wildtype B cells, radiation induces p53-mediated apoptosis and DNA repair while suppressing the target genes of MYC and E2F1, and leads to growth and cell cycle arrest. iDEP helps unveil the multifaceted functions of p53 and the possible involvement of several microRNAs such as miR-92a, miR-504, and miR-30a. In both examples, we validated known molecular pathways and generated novel, testable hypotheses.

**Conclusions:**

Combining comprehensive analytic functionalities with massive annotation databases, iDEP (http://ge-lab.org/idep/) enables biologists to easily translate transcriptomic and proteomic data into actionable insights.

**Electronic supplementary material:**

The online version of this article (10.1186/s12859-018-2486-6) contains supplementary material, which is available to authorized users.

## Background

RNA sequencing (RNA-Seq) [1] has become a routine technique for genome-wide expression analysis. At increasingly reduced cost, library construction and sequencing can often be carried out following standard protocols. For many researchers, especially those without bioinformatics experience, the bottleneck to fully leverage the power of the technique is how to translate expression profiles into actionable insights. A typical analytic workflow involves many steps, each requiring different tools. It can be time-consuming to learn, tune and connect these tools correctly. Another hurdle is the scattered annotation databases with diverse types of gene IDs. To mitigate these issues, we aim to develop an application that can greatly reduce the time and effort required for researchers to analyze RNA-Seq data.

RNA-Seq data analysis often starts with quality control, pre-processing, mapping and summarizing of raw sequencing reads. We assume these steps were completed, using either the traditional Tuxedo Suite [2, 3] or alternatives such as the faster, alignment-free quantification methods [4, 5]. These tools can be used in stand-alone mood or through platforms like GenePattern [6], Galaxy [7], and CyVerse [8].

After read mapping, we often obtain a matrix of gene-level read counts or normalized expression levels (Fragments Per Kilobase Million, or FPKM). For such tabular data, like DNA microarray data, R is a powerful tool for visualization and statistical analysis. In addition, many dedicated R and Bioconductor [9] packages have been developed to identify differentially expressed genes (DEGs) and altered pathways. Some of the packages, such as DESeq2 [10], are developed specifically for the statistical modeling of read counts, and are widely used. But these packages can be time-consuming, or even out of reach for researchers without coding experience.

Several web applications have been developed to analyze summarized expression data (Table 1). START App (Shiny Transcriptome Analysis Resource Tool) is a Shiny app that performs hierarchical clustering, principal component analysis (PCA), gene-level boxplots, and differential gene expression [11]. Another similar tool, Degust [12] can perform differential expression analysis using EdgeR [13] or limma-voom [14] and interactively plot the results. Other tools include Sleuth [15] and ShinyNGS [16]. Non-Shiny applications were also developed to take advantage of the R code base. This includes DEIVA [17] and VisRseq [18]. Beyond differential expression, several tools incorporate some capacity of pathway analysis. For quantified expression data, ASAP (Automated Single-cell Analysis Pipeline) [19] can carry out normalization, filtering, clustering, and enrichment analysis based on Gene Ontology (GO) [20] and KEGG [21] databases. With EXPath Tool [22], users can perform pathway search, GO enrichment and co-expression analysis. Several other Shiny-based tools, such as IRIS [23], are also being developed. The development of these tools in the last few years facilitated the interpretation of RNA-Seq data.

**Table 1:** Comparison of applications for analyzing RNA-Seq

	START App	Degust	ShinyNGS	DEIVA	VisRseq	ASAP	EXPath Tool	IRIS	iDEP
Heatmap	O		O		O	O		O	O
PCA/MDS	O	O	O		O	O		O	O
Clustering of genes			O	O	O			O	O
Volcano/MA Plot	O	O	O	O	O			O	O
Single gene plot	O	O			O				O
Diff. gene expression	O	O	O			O	O	O	O
Co-expression							O		O
Stand-alone R code		O	O			O			O
Pathway analysis		KEGG	KEGG			human& mouse	O		O
Gene ID conversion									O
API to KEGG, STRING-db									O
Complex models								O	O

Note: “O” indicates the functionality is included in a tool.

In this study, we seek to develop a web application with substantially enhanced functionality with (1) automatic gene ID conversion with broad coverage, (2) comprehensive gene annotation and pathway database for both plant and animals, (3) several methods for in-depth EDA and pathway analysis, (4) access to web services such as KEGG [21] and STRING-db [24] via application programming interface (API), and (5) improved reproducibility by generating R scripts for stand-alone analysis.

**Fig. 1:**
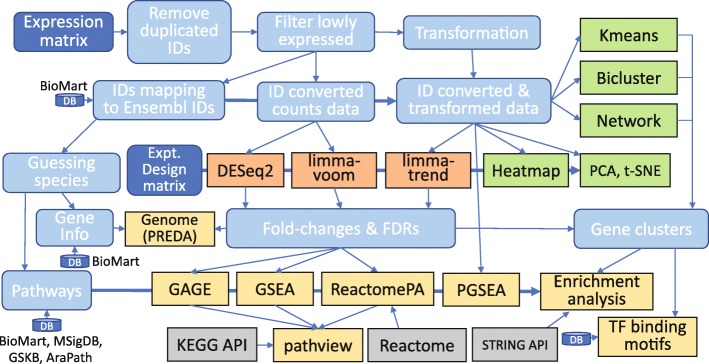
iDEP workflow and functional modules

We used iDEP to analyze two example datasets and generate all the figures and tables in this paper except Table 1 and Fig. 1. We first extensively analyzed a simple RNA-Seq dataset involving small interfering RNA (siRNA)-mediated Hoxa1 knockdown in human lung fibroblasts [3]. We identified the down-regulation of cell-cycle genes, in agreement with previous studies. Our analyses also reveal the possible roles of E2F1 and its target genes, including microRNAs, in blocking G_1_/S transition, and the upregulation of genes related to cytokines, lysosome, and neuronal parts. The second dataset was derived from an experiment with a factorial design to study the effect of ionizing radiation (IR) on mouse B cells with and without functional p53 [25]. In addition to correctly identifying p53 pathway and the enrichment of p53 target genes, we also found the p53-independent effects, including the regulation of ribosome biogenesis and non-coding RNA metabolism, and activation of c-MYC. These examples show that users can gain insights into both molecular pathways and gene regulatory mechanisms.

## Results

We developed an easy-to-use web application for in-depth analysis of gene expression data. iDEP (integrated Differential Expression and Pathway analysis) encompasses many useful R and Bioconductor packages, vast annotation databases, and related web services. The input is a gene-level expression matrix obtained from RNA-seq, DNA microarray, or other platforms. Main functionalities include (1) pre-processing, (2) exploratory data analysis (EDA), (3) differential expression, and (4) pathway analysis and visualization.

Leveraging many existing R packages (see Fig. 1) and the power of the Shiny framework, we developed iDEP to enable users to easily formulate new hypotheses from transcriptomic datasets. We also batch downloaded a massive amount of gene annotation information for 220 species (See Additional file 1: Table S1) from Ensembl [26, 27] Ensembl Plants [28], and Ensembl Metazoa. In addition, comprehensive pathway databases for human (Table 2), mouse [29], and arabidopsis [30] were also compiled from many sources to support in-depth pathway analyses.

**Table 2:** Gene set databases collected for enrichment analysis in human. The last column gives the version of the database, secondary source, or the date of access

Type	Subtype/Database name	Ref.	# Gene Sets	Source & Version
Gene Ontology	Biological Process (BP)	[100]	15,796	Ensembl 92
	Cellular Component (CC)		1916	Ensembl 92
	Molecular Function (MF)		4605	Ensembl 92
KEGG	KEGG	[101]	327	Release 86.1
Curated	Biocarta	[102]	249	Whichgenes 1.5 [103]
	EHMN	[104]	55	GeneSetDB [89]
	Panther	[105]	168	1.0.4
	HumanCyc	[106]	240	Pathway Commons V9 [107]
	INOH	[108]	576	Pathway Commons V9
	NetPath	[109]	27	Pathway Commons V9
	PID	[110]	223	Pathway Commons V9
	PSP	[111]	327	Pathway Commons V9
	Recon X	[112]	2339	Pathway Commons V9
	Reactome	[113]	2010	V64
	WikiPathways	[114]	457	June 10, 2018
TF.Target	CircuitsDB	[115]	829	V2012
	ENCODE	[116]	181	V70.0
	Marbach2016	[117]	628	V 1.0
	RegNetwork	[118]	1400	July 1, 2017
	TFacts	[119]	428	Feb. 2012
	ITFP	[120]	1926	tftargets May,2017
	Neph2012	[121]	16,476	tftargets May,2017
	TRED	[122]	131	tftargets May,2017
	TRRUST	[123]	793	V2
miRNA.Targets	CircuitsDB	[115]	140	V. 2012
	MicroCosm	[124]	44	GeneSetDB
	miRDB	[125]	2588	V 5.0
	miRTarBase	[126]	2599	V 7.0
	RegNetwork	[118]	618	V. 2015
	TargetScan	[127]	219	V7.2
MSigDB.Computational	Computational gene set	[128]	858	MSigDB 6.1
MSigDB.Curated	Literature	[86]	3465	MSigDB 6.1
MSigDB.Hallmark	hallmark	[39]	50	MSigDB 6.1
MSigDB.Immune	Immune system	[129]	4872	MSigDB 6.1
MSigDB.Location	Cytogenetic band	[86]	326	MSigDB 6.1
MSigDB.Motif	TF and miRNA Motifs	[49]	836	MSigDB 6.1
MSigDB.Oncogenic	Oncogenic signatures	[86]	189	MSigDB 6.1
PPI	BioGRID	[130]	15,542	3.4.160
	CORUM	[131]	2178	Feb. 17, 2017
	BIND	[132]	3807	Pathway Commons V9
	DIP	[133]	2630	Pathway Commons V9
	HPRD	[134]	7141	Pathway Commons V9
	IntAct	[135]	11,991	Pathway Commons V9
Drug	MATADOR	[136]	266	GeneSetDB
	SIDER	[137]	473	GeneSetDB
	STITCH	[138]	4616	GeneSetDB
	T3DB	[139]	846	GeneSetDB
	SMPDB	[140]	699	Pathway Commons V9
	CTD	[141]	8758	Pathway Commons V9
	Drugbank	[142]	2563	Pathway Commons V9
Other	CancerGenes	[143]	23	GeneSetDB
	MethCancerDB	[144]	21	GeneSetDB
	MethyCancer	[145]	54	GeneSetDB
	MPO	[146]	3134	GeneSetDB
	HPO	[147]	6785	May, 2018
Total:			140,438	

Our goal was to develop an intuitive, graphical, and robust tool so that researchers without bioinformatics experience can routinely and quickly translate expression data into novel hypotheses. We also wanted to make an open system where users can download intermediate results so that other tools can be used. Also, users can upload custom pathway databases for unannotated species. For experienced bioinformaticians, it can serve as a tool for preliminary analysis as it circumcises the need for many tedious tasks such as converting gene IDs and downloading software packages and annotations. These users can also download customized R scripts and related data files so that the analysis can be reproduced and extended.

### Use case 1: A simple experiment on Hoxa1 knockdown

We first analyzed a simple dataset studying the effect of Hoxa1 knockdown by siRNA in human lung fibroblasts [3]. With 3 replicates for each of the two biological samples, this RNA-Seq dataset was used as example data for the Cuffdiff2 paper [3]. Available as Additional file 2, the read count data was previously used in a tutorial for pathway analysis [31]. A flowchart for the analysis can be found in Additional file 3: Figure S1.

#### Pre-processing and EDA

**Fig. 2:**
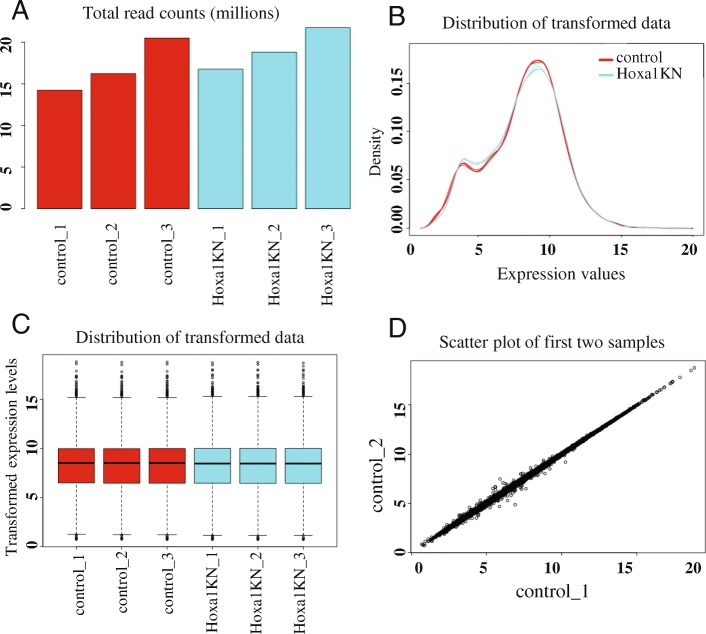
Diagnostic plots for read-counts data. **a** Total read-counts per library. **b** Distribution of transformed data using a density plot. **c** Boxplot of transformed data. **d** Scatter plot of the first two samples

After uploading the read count data, iDEP correctly recognized *Homo sapiens* as the likely species, based on the number of matched genes IDs. After ID conversion and the default filter (0.5 counts per million, or CPM, in at least one sample), 13,819 genes left. A bar plot of total read counts per library is generated (Fig. 2a), showing some small variation in library sizes. We chose the regularized log (rlog) transformation implemented in the DESeq2 package, as it effectively reduces mean-dependent variance (Additional file 3: Figure S2). Distribution of the transformed data is shown in Fig. 2b-c. Variation among replicates is small (Fig. 2d).

**Fig. 3:**
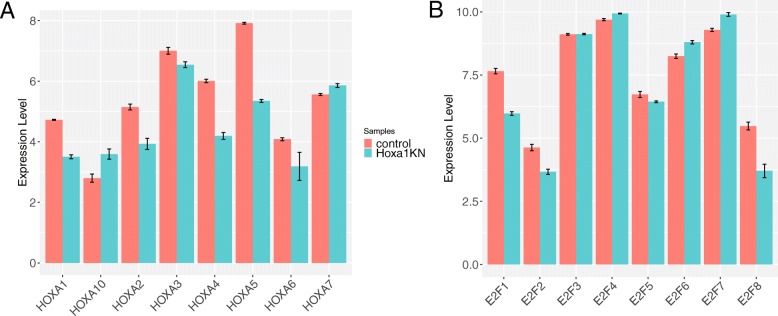
Expression patterns of (**a**) Hoxa and (**b**) E2f gene families generated by iDEP

iDEP also enables users to examine the expression level of one or more genes. Using “Hoxa” as a keyword, we obtained Fig. 3a, which shows that Hoxa1 expression level is reduced, but not abolished, in response to siRNA-mediated knockdown of Hoxa1. Noticeably, expression of other family members, especially Hoxa2, 4, and 5, also decrease. As these genes have similar mRNA sequences, it is unclear whether this is caused by off-target effects of the siRNA or ambiguous mapping of RNA-Seq reads. Figure 3b, obtained by using “E2F” as a keyword, shows the down-regulation of E2F1.

**Fig. 4:**
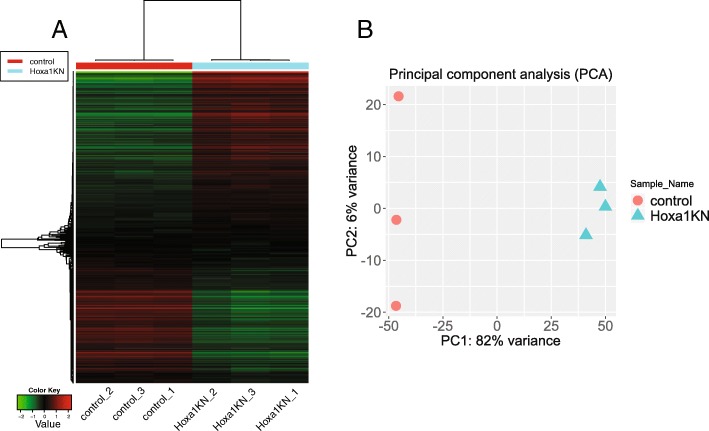
Hierarchical clustering (**a**) and PCA analyses (**b**) indicate the substantial difference in thousands of genes induced by Hoxa1 knockdown. There is little variation among replicates

We rank genes by their standard deviation across all samples and use the top 1000 genes in hierarchical clustering. The result in Fig. 4a suggests that Hoxa1 knockdown in lung fibroblast cells induce a drastic change in the expression of hundreds of genes. Variations among technical replicates are minimal. These observations can also be confirmed by the correlation matrix (Additional file 3: Figure S3) and k-means clustering (Additional file 3: Figure S4).

PCA plot using the first and second principal components is shown in Fig. 4b. There is a clear difference between the Hoxa1 knockdown and the control samples, along the first principal component that explains 93% of the variance. Plot using multidimensional scaling (MDS), and t-SNE [32] also show a similar distribution of the samples (Additional file 3: Figure S5). We can choose to conduct pathway analysis using PGSEA [33, 34] by treating the loadings of the principal components as expression values. As suggested by Additional file 3: Figure S6, the first two components are related to cell cycle regulation.

#### Differentially expressed genes (DEGs)

**Fig. 5:**
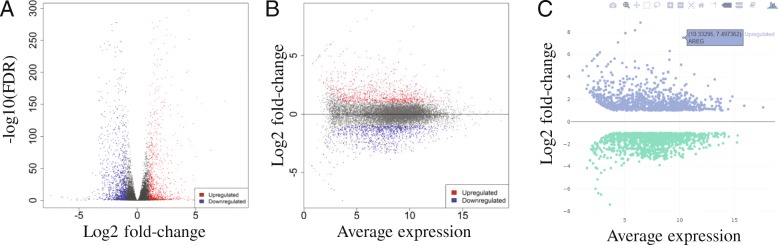
Summary plots for differential expression analysis using DESeq2. **a** Volcano plot, **b** MA plot, and (**c**) Interactive MA plot

With the DESeq2 package, we identified 907 upregulated and 1097 downregulated genes (see Additional file 1: Table S3) using a threshold of false discovery rate (FDR) < 0.1 and fold-change > 2. The volcano plot (Fig. 5a) and the MA plot (Fig. 5b) show that Hoxa1 knockdown leads to a massive transcriptomic response. Plotly-based interactive versions of these plots are also available, where users can zoom in and mouse over to see individual genes (Fig. 5c). A quick scan at the top genes ranked by the absolute values of fold-change (FCs) tells us that Hoxa1 knockdown induces cytokines (IL1B, IL24).

The up and down-regulated genes are then subjected to enrichment analysis based on the hypergeometric distribution. Many different types of genes sets listed in Table 2 can be used to test various hypotheses. The GO Biological Process terms enriched in DEGs are shown in Table 3. Upregulated genes are related to regulation of cell proliferation, locomotion, and response to endogenous stimuli. This is perhaps the cell’s response to injected siRNAs. The downregulated genes are significantly enriched with cell cycle-related genes (FDR < 2.6 × 10^− 47^). The effect of Hoxa1 knockdown on cell cycle was reported and experimentally confirmed in the original study [3]. Cell cycle analysis revealed that loss of Hoxa1 leads to a block in G_1_ phase [3].

**Table 3:** Enriched GO terms in up and down-regulated genes

Direction	Pathways	nGenes	adj.Pval
Down Regulated	Cell cycle	259	4.50E-46
	Cell cycle process	207	1.70E-42
	Mitotic cell cycle	169	5.60E-38
	Chromosome segregation	92	2.80E-35
	Mitotic cell cycle process	145	2.00E-33
	Sister chromatid segregation	72	5.20E-33
	Cell division	118	5.20E-33
	Nuclear chromosome segregation	80	2.20E-30
	Nuclear division	86	5.60E-26
	Organelle organization	350	1.20E-24
	Mitotic nuclear division	65	3.50E-24
	Organelle fission	88	5.80E-24
	Cytoskeleton organization	159	1.70E-23
	Cell cycle phase transition	98	2.60E-21
	Mitotic sister chromatid segregation	45	4.00E-21
Up Regulated	Cell surface receptor signaling pathway	258	3.40E-25
	Regulation of cell proliferation	171	8.20E-23
	Cell proliferation	195	7.40E-22
	Regulation of signaling	265	3.80E-21
	Response to organic substance	259	1.20E-20
	Regulation of cell communication	260	1.20E-20
	Locomotion	165	1.20E-19
	Regulation of signal transduction	239	1.30E-19
	System development	332	1.00E-18
	Regulation of cellular component movement	100	3.70E-17
	Regulation of response to stimulus	291	8.20E-17
	Response to endogenous stimulus	143	1.20E-16
	Response to chemical	319	1.70E-16
	Cellular response to organic substance	211	1.80E-16
	Cell migration	131	2.10E-16

**Fig. 6:**
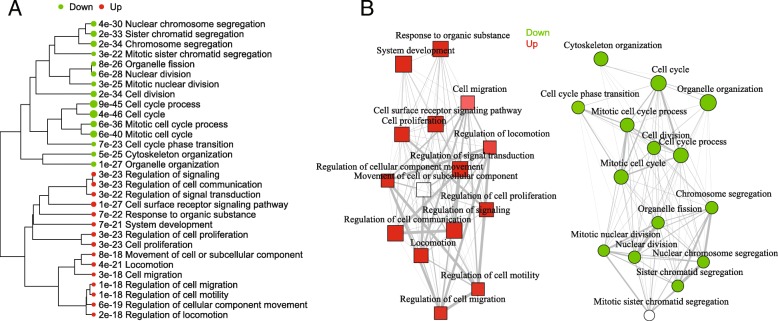
Visualization of the relationship among enriched GO terms using (**a**) hierarchical clustering tree and (**b**) network

As many GO terms are related or redundant (i.e.*,* cell cycle and cell cycle process), we provide two plots to summarize such correlation [35]. We first measure the distance among the terms by the percentage of overlapped genes. Then this distance is used to construct a hierarchical clustering tree (Fig. 6a) and a network of GO terms (Fig. 6b). Both plots show that the enriched terms are distinct in the two gene lists. The down-regulated genes are overwhelmingly involved in cell cycle. The upregulated genes are related to 4 related themes: cell proliferation, signaling, response to organic substance, and cell migration, possibly in reaction to the injected siRNAs.

Choosing GO cellular component, we find that Hoxa1 knockdown suppresses genes that code for the spindle, cytoskeleton and chromosomal parts (Additional file 3: Figure S7). As Hoxa1 knockdown blocks G_1_/S transition [3], a smaller number of cells are in the S (synthesis) phase, leading to the reduction of proteins related to the spindle and chromosomal parts. Hoxa1 knockdown also induces genes related to plasma membrane, neurons and synapses (Additional file 3: Figure S7). This unexpected result is consistent with Hoxa1’s role in neuronal differentiation [36, 37]. Polymorphisms of this gene are associated with cerebellar volume in humans [38]. Hoxa1 may have different functions in various organs across developmental stages.

Choosing KEGG pathway, we confirm the overrepresentation of cell cycle-related genes in downregulated genes (Additional file 3: Figure S8). For up-regulated genes, we detect cytokine-cytokine receptor interaction (CCRI) pathway (FDR < 1.3 × 10^− 10^). “MSigDB.Curated” gene sets contain pathways from various databases as well as published lists of DEGs from previous expression studies [39]. As shown in Additional file 3: Figure S9, the most significant are oligodendrocyte differentiation and several cell-cycle related gene sets. As suggested by a meta-analysis of published gene lists [40], cell-cycle related expression signature is frequently triggered by diverse cellular perturbations [41]. We uncovered similarity of our expression signature with previously published ones.

**Fig. 7:**
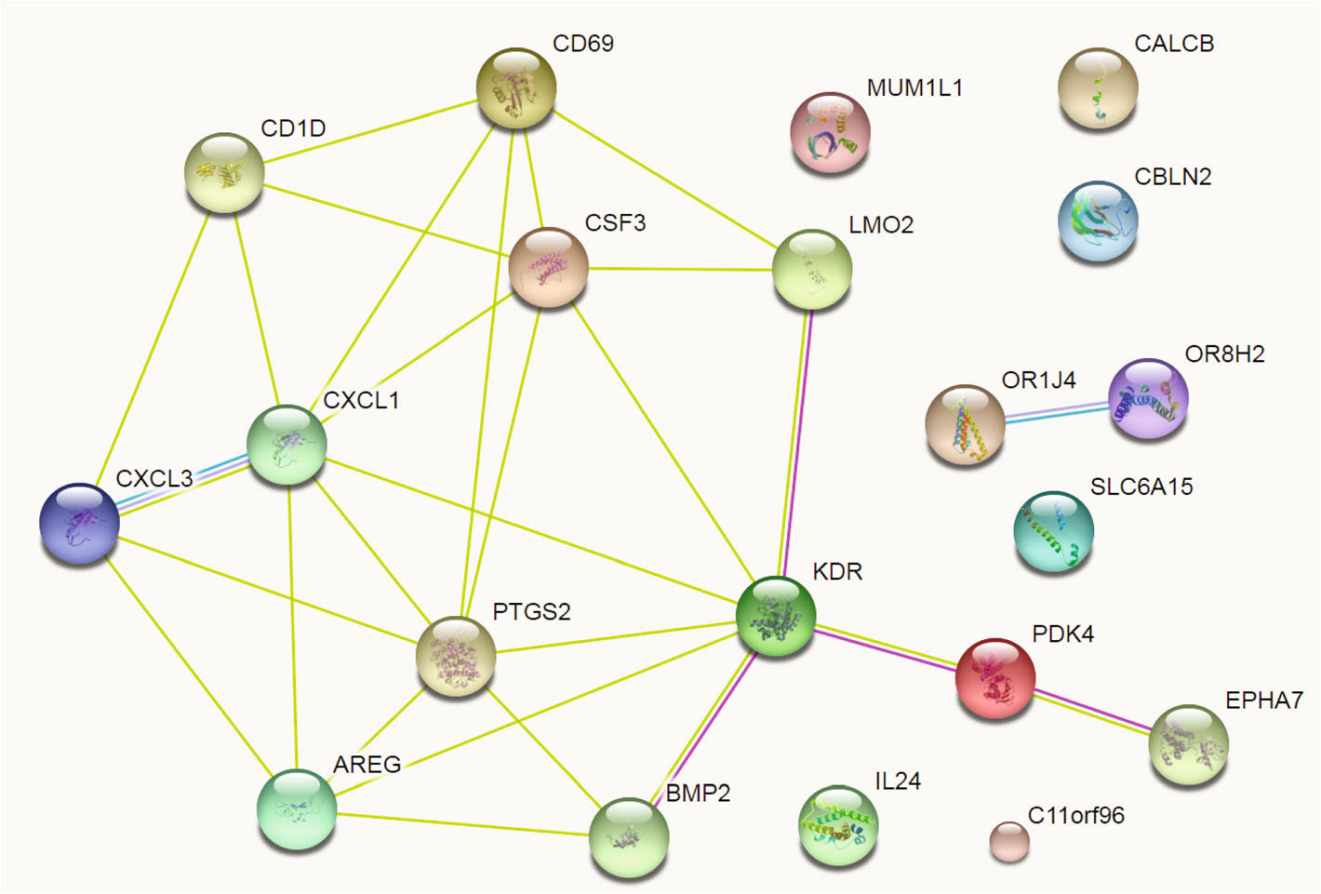
Protein-protein interactions (PPI) among top 20 up-regulated genes. This is retrieved via API access to the STRING database. There is also an enrichment of PPIs compared with background. An interactive, annotated version of this network is also available through a link to the STRING website

Using the STRINGdb package, iDEP can analyze the lists of DEGs via the STRING API [24] for enrichment analysis and the retrieval of PPI networks. The enrichment analysis led to similar results (Additional file 1: Table S4) to those obtained using the internal iDEP gene sets. In addition, STRING detected that the Helix-loop-helix DNA-binding domain is overrepresented in proteins coded by the 907 upregulated genes, while the Tubulin/FtsZ family, GTPase domain is enriched in the 1097 down-regulated genes (Additional file 1: Table S5). Figure 7 is the network of PPIs among the top 20 upregulated genes. The highly connected network includes chemokine ligands 1 and 3 (CXCL1 and CXCL3), as well as interleukin 24 (IL24), suggesting the immune response caused by injected siRNA. A link to an interactive, richly annotated version of this network on the STRING website is also available.

iDEP can also reveal gene regulatory mechanisms. Using the transcription factor (TF) target gene sets in enrichment analyses, we can obtain Table 4, which suggest that target genes of SP1 (FDR < 9.80 × 10^− 23^) and E2F factors (FDR < 1.1 × 10^− 16^) are overrepresented in down-regulated genes. E2F factors are regulators of cell cycle [42]. E2F1 promotes G_1_/S transition [43] by regulation many genes, including itself. SP1 binding sites were identified in cell-cycle related genes such as Cyclin D1 (CCD1) [44]. SP1 is a G1 phase specific TF [45]. The interaction of E2F1 and SP1 proteins mediate cell cycle regulation [46]. The upregulated genes are enriched with target genes of NF-κB (FDR < 4.9 × 10^− 9^) and FOXO3 (FDR < 4.9 × 10^− 9^), known regulators of the immune response [47, 48].

**Table 4:** Enriched transcription factor (TF) binding motifs

Direction	Pathways	nGenes	adj.Pval
Down Regulated	Tftargets:TF Target SP1	475	9.80E-23
	Tftargets:TF Target E2F-4	65	9.80E-23
	TFactS E2F1	43	1.10E-16
	TRRUST:TF Target E2F1	37	9.70E-15
	RegNetwork:TF Target E2F4	127	1.20E-14
	RegNetwork:TF Target E2F1	208	1.00E-13
	TFactS E2F4	20	1.30E-12
	RegNetwork:TF Target NFYA	154	4.60E-09
	TFactS E2F3	15	1.80E-08
	Tftargets:TF Target TEAD1	33	1.90E-08
	Tftargets:TF Target AP1	74	2.30E-08
	TFactS E2F2	14	2.60E-08
	Tftargets:TF Target TGIF1	34	3.30E-08
	Tftargets:TF Target ZNF219	60	4.50E-08
	Tftargets:TF Target HF1H3B	101	5.00E-08
Up Regulated	Tftargets:TF Target NFKB	54	4.90E-09
	TFactS FOXO3	21	4.90E-09
	Tftargets:TF Target NFKB1	34	7.30E-09
	TRRUST:TF Target NFKB1	42	7.30E-09
	TFactS CTNNB1	40	4.40E-08
	Tftargets:TF Target FOXJ1	22	2.00E-07
	Tftargets:TF Target POU3F2	35	4.00E-07
	TRRUST:TF Target RELA	38	4.20E-07
	Tftargets:TF Target FOXO3	28	4.80E-07
	TRRUST:TF Target SP1	50	4.80E-07
	TRRUST:TF Target JUN	25	5.20E-07
	TRRUST:TF Target EGR1	19	6.10E-07
	Tftargets:TF Target SP1	39	1.20E-06
	Tftargets:TF Target FOXJ3	25	1.20E-06
	Tftargets:TF Target FOXL1	24	1.20E-06

**Fig. 8:**
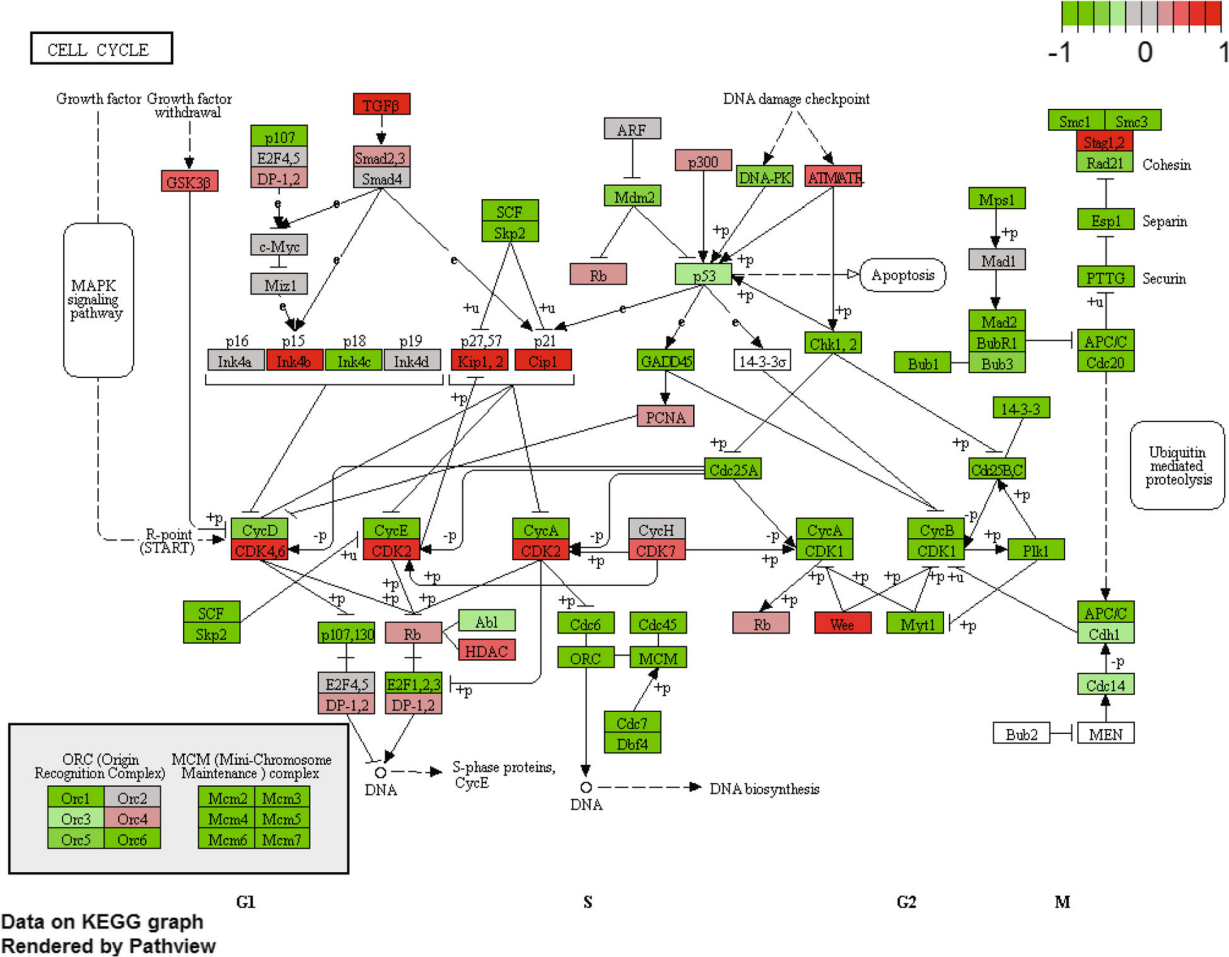
Expression profiles of cell-cycle related genes visualized on an KEGG pathway diagram using the Pathview package. Red and green indicate genes induced or suppressed by Hoxa1 knockdown, respectively

The Motif gene sets from MSigDB are derived from [49] and contain sets of genes sharing TF binding motifs in gene promoters and microRNA target motifs in 3′ untranslated regions (UTRs). Using this gene set, we again detect the enrichment of E2F motifs in promoters of downregulated genes (Additional file 1: Table S16). We also detected overrepresentation of a “GCACTTT” motif in 3’ UTRs of upregulated genes. This motif is targeted by several microRNAs, namely miR-17-5P, miR-20a, miR-106a. Cloonan et al. showed that miR-17-5P targets more than 20 genes involved in the G_1_/S transition [30]. Trompeter et al. provided evidence that miR-17, miR-20a, and miR-106b enhance the activities of E2F factors to influence G_1_/S transition [50]. miR-106b resides in the intron of Mcm7 along the sense direction. Mcm7 is an E2F1 target gene that is also downregulated by Hoxa1 knockdown (see Fig. 8a). Petrocca et al. showed that E2F1 regulates miR-106b, which can conversely control E2F1 expression [51]. Thus, it is possible that Hoxa1 knockdown reduces E2F1 expression (see Fig. 3b) and its target genes, including Mcm7, which hosts miR-106b. We can speculate that downregulated miR-106b, in turn, causes the increases in the expression of its target genes. Leveraging the comprehensive pathway databases, iDEP enables researchers to develop new hypotheses that could be further investigated.

For many species, predicted TF target genes are not available. We downloaded 300 bp and 600 bp promoter sequences from ENSEMBL and scanned them with a large collection of TF binding motifs [52]. As shown in Table 5, the promoters of DEGs are overrepresented with many G-rich motifs bound by E2F and other factors such as TCFL5 and SP2. We compared the best possible scores for each TF and promoter pair and run t-tests to compare these scores. Further study is needed to validate this approach.

**Table 5:** TF motifs enriched in gene promoters (300 bp) of up- or down-regulated genes

List	Motif	TF	TF family	FDR
Down Regulated	GGCGGGAA	E2F4	E2F	3.40E-14
	GGCCGGAG	MBD2	MBD	7.70E-14
	CACGTG	TCFL5	bHLH	2.30E-11
	GGGGGCGGGGC	SP2	C2H2 ZF	3.40E-11
	GGGCGGGAA	E2F6	E2F	8.60E-10
	GTGGGCGTGGC	SP6	C2H2 ZF	2.10E-09
	TGCGGG	ZBTB1	C2H2 ZF	2.20E-08
	GGGCGTG	KLF7	C2H2 ZF	2.90E-08
	ATGCGTGGGCGG	EGR4	C2H2 ZF	1.50E-07
	CACAGCGGGGGGTC	ZIC4	C2H2 ZF	1.80E-07
Up Regulated	GGGGGCGGGGC	SP2	C2H2 ZF	2.20E-06
	GGGGGGGGGCC	PATZ1	C2H2 ZF	2.40E-06
	TGCGGG	ZBTB1	C2H2 ZF	2.60E-06
	GGGGGGT	ZIC5	C2H2 ZF	1.40E-04
	GGCCGGAG	MBD2	MBD	1.50E-04
	CACGTG	TCFL5	bHLH	1.50E-04
	CACAGCGGGGGGTC	ZIC4	C2H2 ZF	1.10E-03
	GGGGCCCAAGGGGG	PLAG1	C2H2 ZF	1.10E-03
	GTGGGCGTGG	SP8	C2H2 ZF	1.40E-03
	GTGGGCGTGGC	SP6	C2H2 ZF	2.30E-03

For human (Table 2), mouse [29] and Arabidopsis [53], we also include predicted target genes for many miRNAs from multiple sources. Using these gene sets, we detected significant enrichment (Table 6) of miRNA-193b, miR-192, and miR-215 target genes among the down-regulated genes. miR-193b was shown to suppress cell proliferation and down-regulate CCND1 [54]. Proposed as biomarkers of several cancers, miR-192 also inhibit proliferation and can cause cell cycle arrest when overexpressed [55]. miR-215 shares many target genes with miR-192 and are also downregulated in cancer tissues [56]. These miRNAs may play a role in the regulation of cell cycle upon Hoxa1 knockdown.

**Table 6:** Enriched miRNA target gene sets

Direction	adj.Pval	nGenes	Pathways
Down Regulated	3.40E-45	162	MiRTarBase:miRNA Target hsa-miR-193b-3p
	1.50E-41	170	MiRTarBase:miRNA Target hsa-miR-192-5p
	4.20E-41	145	MiRTarBase:miRNA Target hsa-miR-215-5p
	6.20E-18	162	MiRTarBase:miRNA Target hsa-miR-124-3p
	3.30E-08	82	MiRTarBase:miRNA Target hsa-miR-34a-5p
	9.40E-07	66	MiRTarBase:miRNA Target hsa-miR-7-5p
	9.40E-07	58	MiRTarBase:miRNA Target hsa-miR-375
	3.00E-06	85	MiRTarBase:miRNA Target hsa-miR-24-3p
	4.60E-06	89	MiRTarBase:miRNA Target hsa-miR-1-3p
	6.80E-05	84	MiRTarBase:miRNA Target hsa-miR-155-5p
Up Regulated	6.00E-16	206	MiRTarBase:miRNA Target hsa-miR-335-5p
	2.30E-12	99	RegNetwork:miRNA Target hsa-miR-144
	1.50E-10	81	RegNetwork:miRNA Target hsa-miR-29c
	3.30E-10	81	RegNetwork:miRNA Target hsa-miR-29b
	3.30E-10	100	RegNetwork:miRNA Target hsa-miR-93
	3.90E-09	72	RegNetwork:miRNA Target hsa-miR-29a
	6.20E-09	94	RegNetwork:miRNA Target hsa-miR-30e
	6.20E-09	101	RegNetwork:miRNA Target hsa-miR-340
	6.20E-09	67	RegNetwork:miRNA Target hsa-miR-519d
	5.70E-08	46	RegNetwork:miRNA Target hsa-miR-17-5p

#### Pathway analysis

Instead of using selected DEGs that are sensitive to arbitrary cutoffs, pathway analysis can use fold-change values of all genes to identify coherently altered pathways. We used the GAGE (generally applicable gene set enrichment) [57] as a method and KEGG as gene sets. The results (Additional file 1: Table S6) is similar to those from a previous analysis by Turner in an online tutorial [31] and also agrees with our enrichment analysis based on DEGs. For each of the significant KEGG pathways, we can view the fold-changes of related genes on a pathway diagram using the Pathview Bioconductor package [58]. Many cell cycle genes are marked as green in Fig. 8, indicating reduced expression in Hoxa1-knockdown samples. We also detected upregulation of genes related to CCRI, arthritis, and lysosome. Many CCRI related genes are up-regulated (Additional file 3: Figure S10). Not detected using DEGs, lysosome-related genes are mostly upregulated (Additional file 3: Figure S11). Injected siRNAs might be degraded in the lysosome.

**Fig. 9:**
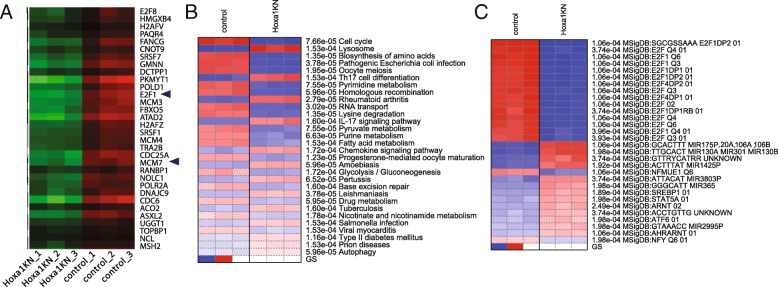
Pathway analysis results using different options. **a** expression patterns of genes with E2F1 binding motifs. E2F1 gene itself is also downregulated in Hoxa1 knockdown. So is the Mcm7 gene, whose intron host miR-106b-25 clusters. **b** Results from running PGSEA on KEGG gene sets. **c** PGSEA applied on MSigDB.Motif gene sets

By changing the gene sets database for pathway analysis, we can gain further insights. Using MSigDB.Motif gene sets, we can verify the enrichment of E2F binding motifs (Additional file 1: Table S7). For non-KEGG gene sets, heatmaps are created to show the expression of genes in significant gene sets. Figure 9a shows part of such a plot, highlighting genes that share the “SGCGSSAAA” motif bound by E2F1. Note that E2F1 gene itself is included in the figure, as it binds to its own promoter and forms a positive feedback loop [43]. The downloaded expression data indicate that E2F1 is downregulated by more than 3-fold in Hoxa1 knockdown samples (see Fig. 3b). Upon Hoxa1 knockdown, downregulation of E2F1 and downstream genes, including microRNAs, may be part of the transcription program that blocks G_1_/S transition.

Users can use many combinations of methods and gene sets to conduct pathway analysis. For example, using PGSEA on KEGG pathways yielded Fig. 9a and b, again confirming previous results on suppressed cell cycle genes and induced lysosome and CCRI related genes. Using the MSigDB.Motif gene sets, we can also confirm the E2F1 binding motifs (Fig. 9). The most highly activated gene sets are related to miR-17-5p, miR-20a, miR106a,b and so on (Fig. 9c), which agrees with enrichment analysis using just gene lists.

Some pathways can be attenuated by upregulating some of its associated genes while downregulating others. To detect such pathways, we can use the absolute values of fold changes in pathway analysis. This is achieved by checking the box labeled “Use absolute values of fold changes for GSEA and GAGE.” Instead of detecting up or down-regulated pathways, the results show which pathways are more regulated. As shown in Additional file 1: Table S8, while the expression of ribosome related genes is less variable upon Hoxa1 knockdown, genes related to CCRI are highly regulated.

**Fig. 10:**
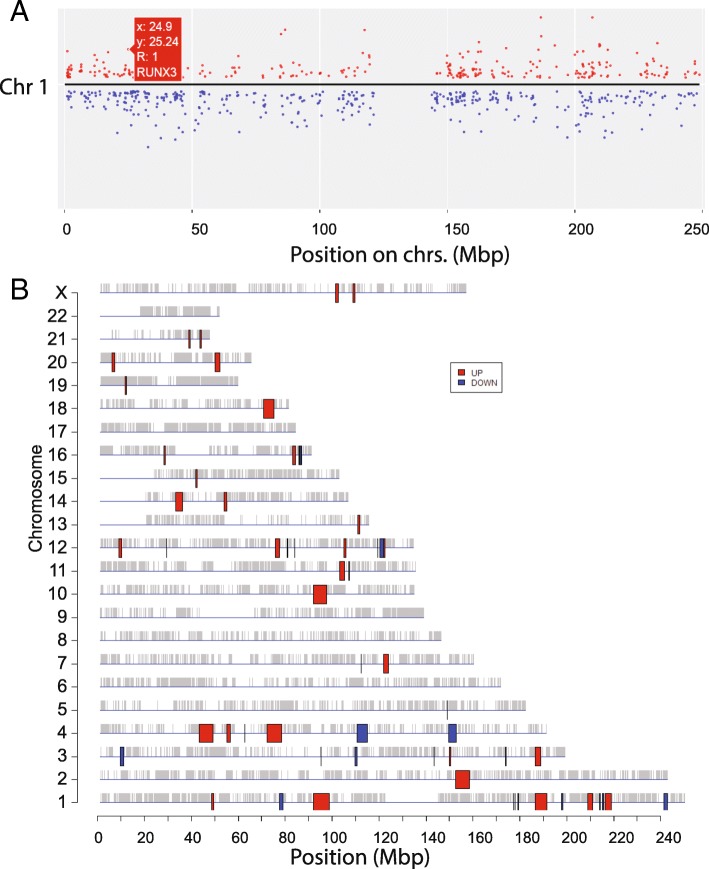
Visualizing expression profiles on chromosomes. **a** Zoom-in on Chr. 1 using the dynamic graphics, showing the upregulation of RUX3 gene. **b** Statistically significant genomic regions identified by PREDA

The expression of neighboring genes can be correlated due to mechanisms such as super-enhancers [59], 3D chromatin structure [60], or genomic gain or loss in cancer. To help users detect such correlation, we use ggplot2 [61] and Plotly to interactively visualize fold-changes on all the chromosomes (Fig. 10a). Based on regression analysis, the PREDA package [62] can detect statistically significant chromosomal regions with coherent expression change among neighboring genes. Figure 10b shows many such regions in response to Hoxa1 knockdown. Detailed information obtained from downloaded files (Additional file 1: Table S9) suggests, for example, a 4.3 Mbps region on Chr.1q31 contains 6 upregulated genes (PRG4, TPR, C1orf27, PTGS2, PLA2G4A, and BRINP3).

To further validate our parameterization of PREDA, we analyzed DNA microarray data (Additional file 4) of thymus tissues from patients with Down syndrome [63]. We detected large, upregulated regions on chromosome 21 (Additional file 3: Figure S12), as expected. Even though PREDA analysis is slow and has low-resolution due to the use of gene-level expression score, it might be useful in cancer studies where localized expression change on the chromosome can happen.

To improve reproducibility, iDEP generates custom R code and R Markdown code based on user data and choices of parameters (Additional files 5, 6 and 7). Users with some R coding experience should be able to re-run most analyses by downloading the related annotation and gene sets used by iDEP. An example is shown here [64].

### Use case 2: p53’s role in response to ionizing radiation

Tonelli et al. [25] used RNA-Seq to study the effect of whole-body ionizing radiation (IR) on the mouse with or without p53. B cells and non-B cells were isolated from mouse spleen after treatment. We analyzed the B cell data involving two genotypes (p53 wildtype and p53 null) with mock or IR treatment, a typical 2 × 2 factorial design. The read count and experimental design files are available as Additional files 8 and 9. A converted, filtered version of this dataset is incorporated into iDEP as a demo data.

With this dataset, we demonstrate how users can easily generate hypothesis on molecular pathways and gene regulatory mechanisms through three steps: (1) enrichment analysis of k-means clusters, (2) enrichment analysis of the lists of DEGs, and (3) pathway analysis using fold-changes values of all genes.

#### Pre-process and EDA of p53 dataset

**Fig. 11:**
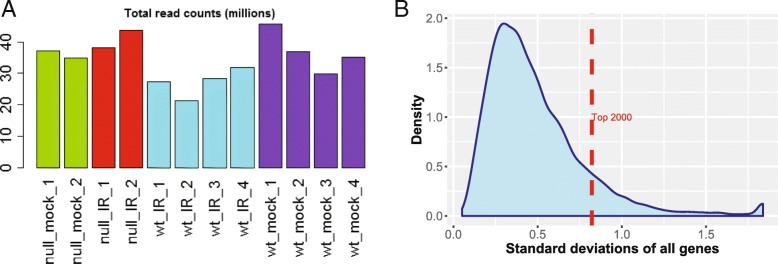
**a** Total read counts are smaller in the WT samples treated with IR. **b** Distribution of standard deviations for all genes

We noticed reduced total reads for wildtype samples treated with IR (Fig. 11a). While this may be caused by biology, but biased sequencing depth presents a confounding factor, that has not been discussed widely. To quantify such biases, iDEP routinely performs ANOVA analysis of total read counts across sample groups. For this example, uneven read counts are detected (*P* = 0.047) and a warning is produced.

EDA shows that IR treatment led to the changes in thousands of genes. Based on the distribution of variances (Fig. 11b), we choose the top 2500 genes for clustering analysis. Hierarchical clustering (Additional file 3: Figure S13) shows the substantial differences between treated and untreated samples. It also shows the patterns of different groups of genes and the variations among some replicates of treated wild-type cells (wt_IR).

**Fig. 12:**
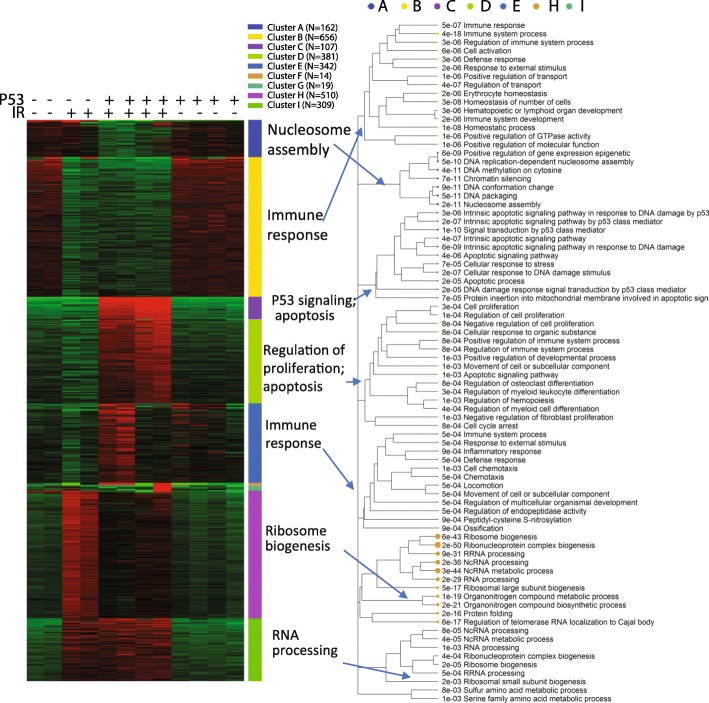
K-means clustering and enrichment analysis of the ionization dataset

We then used k-means clustering to divide the top 2500 genes into groups. Based on the within-group sum of squares plot (Additional file 3: Figure S14) as a reference, we chose a slightly larger k = 9. Figure 12 shows the 9 gene clusters and the enriched GO terms. Details are available in Additional file 1: Tables S10 and S11. Genes in clusters B and I show similar responses to IR across genotypes. Strongly enriched in genes related to the immune system (FDR < 3.65 × 10^− 18^), cluster B are downregulated by IR in both cell types. The immune-suppressive effects of radiation [65] are clearly p53-independent. Induced by IR in both wildtype and Trp53^−/−^ cells, cluster I genes are enriched in ribosome biogenesis but with much lower level of significance (FDR < 2.25 × 10^− 5^).

On the other hand, genes in clusters A, C, and D are specific to the wild-type cells. Cluster A contains 13 genes that code for histone proteins and are involved in nucleosome assembly (FDR < 1.66 × 10^− 11^). Genes in Clusters C and D are induced by IR only in B cells with p53, but the former is more strongly upregulated. As expected, cluster C is related to the p53 pathway (FDR < 1.38 × 10^− 10^) and apoptosis (FDR < 3.59 × 10^− 6^). It is enriched with 15 p53 target genes like Mdm2 (FDR < 3.53 × 10^− 18^). Cluster D genes are related to the regulation of cell proliferation and cell cycle arrest, representing further downstream of the transcriptional cascade of p53 signaling.

Genes in cluster H are more highly upregulated in Trp53^−/−^ B cells than wildtype cells. It is overrepresented with non-coding RNA (ncRNA) processing (FDR < 3.25 × 10^− 36^), ribosome biogenesis (FDR < 5.53 × 10^− 43^), and protein folding (FDR < 2.23 × 10^− 16^). Many of these genes code for proteins in the nucleus and mitochondrion. Significant enrichment of 7 c-Myc target genes is observed (FDR < 5.09 × 10^− 7^). Many of these enrichment results will be further validated in enrichment analysis of DEGs and pathway analysis. Enrichment analysis of the k-Means clusters provides an opportunity to gain insight into the molecular pathways underlying different patterns of gene expression.

#### Identifying DEGs in the p53 dataset

To identify genes induced by IR in both cell types, users can use pair-wise comparisons among the 4 sample groups. Alternatively, we can construct linear models through the GUI. Here we use the following model:

- Expression ~ p53 + Treatment + p53:Treatment,

where the last term represents the interaction between genotype and treatment, capturing the additional effects of p53 in IR response. It is important to set the reference levels for factors in a model. Here we set the null (Trp53^−/−^) as a reference level for the factor “p53” and mock for the factor “Treatment”. More details about statistical models is available [66].

**Fig. 13:**
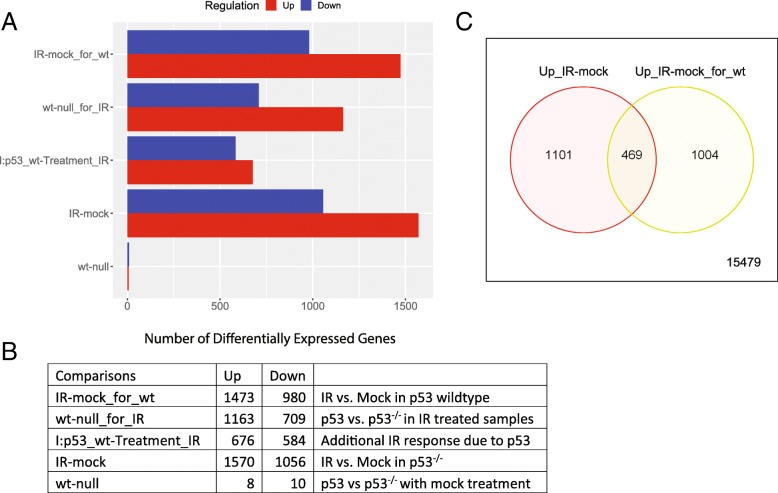
Statistics of DEGs identified by DESeq2. **a** and **b** shows the numbers of differentially expressed genes for each comparison. **c** Venn Diagram shows the overlap between IR induced genes in WT and P53 null samples

With FDR < 0.01 and fold-change > 2 as cutoffs, we used DESeq2 to identify DEGs (Fig. 13a and b). Without treatment, the two cell types have similar transcription profiles, with few DEGs. But even in Trp53^−/−^ cells, IR caused the upregulation of 1570 genes, 469 of which is also upregulated in p53 wildtype B cells (see Venn diagram in Fig. 13c). PPI networks for the top up- and down-regulated genes in wildtype cells are shown in Additional file 3: Figures S15 and S16, respectively.

To further understand the molecular pathways, we perform enrichment analysis of the 10 gene lists (Additional file 1: Table S12) associated with 5 comparisons. We focus on two comparisons (1) “IR-mock” representing the baseline response of IR in mutant cells without p53, and (2) “I:p53_wt-Treatment_IR”, the interaction term capturing the additional effect of p53 compared to the baseline response.

For the first comparison, Additional file 3: Figure S17 shows IR induced DEGs in mutant cells. The 1570 upregulated genes are related to non-coding RNA (ncRNA) metabolic process (FDR < 1.33 × 10^− 79^), ribosome biogenesis (FDR < 2.54 × 10^− 67^), and translation (FDR < 3.03 × 10^− 32^). This enrichment profile is similar to cluster H derived from the k-Means clustering, as the two lists capture the same group of genes. The upregulated genes are surprisingly coherent in function. For example, 219 (14%) can be found in the nucleus, 286 (18%) is related to the mitochondrion, and, most significantly, 407 (26%) is RNA-binding (FDR < 3.54 × 10^− 138^). The 1570 upregulated genes contain 7 MYC target genes (FDR < 4.22 × 10^− 7^), consistent with the fact that MYC is a direct regulator of ribosome biogenesis [67]. This agrees with reports of the involvement of MYC in radiation treatment [68, 69], suggesting MYC may trigger proliferation pathways upon genotoxic stress, in the absence of p53.

Genes downregulated by IR in Trp53^−/−^ B cells are related to immune system (FDR < 4.22 × 10^− 8^), GTPase activity (FDR < 3.75 × 10^− 6^), and actin cytoskeleton (FDR < 2.06 × 10^− 5^). As shown in Additional file 1: Table S13, we can also detect the enrichment of the target genes of miR-124 (FDR < 4.56 × 10^− 12^), an important modulator of immunity [70]. Others associated miRNAs, including miR-6931-5p, Mir-4321, and miR-576-5p, may also be involved.

**Fig. 14:**
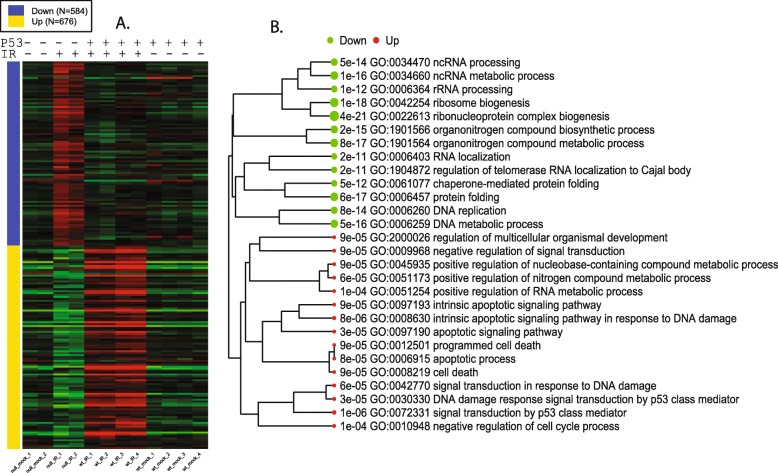
Additional effect of p53 in IR response. **a** Expression patterns of selected DEGs. **b** Upregulated genes are enriched with genes related to p53 mediated response to DNA damage, especially apoptosis, and negative regulation of cell cycle. These genes are only induced by IR in cells with wildtype p53. One the other hand, p53 caused the relative downregulation of genes related to ribosome biogenesis, tRNA and rRNA processing, DNA replication, and protein folding. These genes are only upregulated by IR in Trp53−/−

For the second comparison, the expression profiles of DEGs associated with the interaction term is shown in Fig. 14. This is the p53 mediated IR response, compared to the baseline response without p53. The 676 genes that are upregulated in wild-type B cells following IR, but not in Trp53^−/−^ B cells. As expected, these genes are enriched in p53-mediated response to DNA damage (FDR < 1.43 × 10^− 6^), and apoptosis (FDR < 9.72 × 10^− 6^). As shown in Additional file 1: Table S13, these genes are overrepresented with 25 target genes of p53 (FDR < 1.34 × 10^− 13^) and 76 target genes of miR-92a (FDR < 2.79 × 10^− 11^). Part of the miR-17/92 cluster, miR-92a is related to tumorigenesis and is regulated by p53 [71, 72]. Another miRNA with overrepresented target genes is miR-504 (FDR < 3.25 × 10^− 8^), which has been shown to binds to 3’ UTR of Trp53 and negatively regulate its expression [73]. Located in the introns of the fibroblast growth factor 13 (FGF13) gene, miR-504 is transcriptionally suppressed by p53, forming a negative feedback loop [74]. Following radiation, the expression of both miR-92a and miR-504 in wild-type B cells may be reduced, leading to the upregulation of their target genes. Further study is needed to verify this hypothesis.

As shown in Fig. 14, the 584 genes downregulated according to the interaction term are those that are induced in the Trp53^−/−^ B cells, but not in wild-type B cells. These genes are overrepresented with ncRNA processing, ribosome biogenesis, cell cycle, and RNA transport (Additional file 1: Table S14). Most (411) of the 584 genes are included in the genes upregulated by IR in Trp53^−/−^ B cells, as suggested by the Venn diagram in Additional file 3: Figure S18. MYC target genes are also downregulated by p53 upon IR. In wildtype B cells, p53 suppresses the MYC oncogenic pathway compared to Trp53^−/−^ B cells. The most significant shared TF binding motif is E2F1 (FDR < 7.73 × 10^− 11^). This agrees with the role of p53 in cell cycle arrest through p21-mediated control of E2F factors [75].

#### Pathway analysis of p53 data

**Fig. 15:**
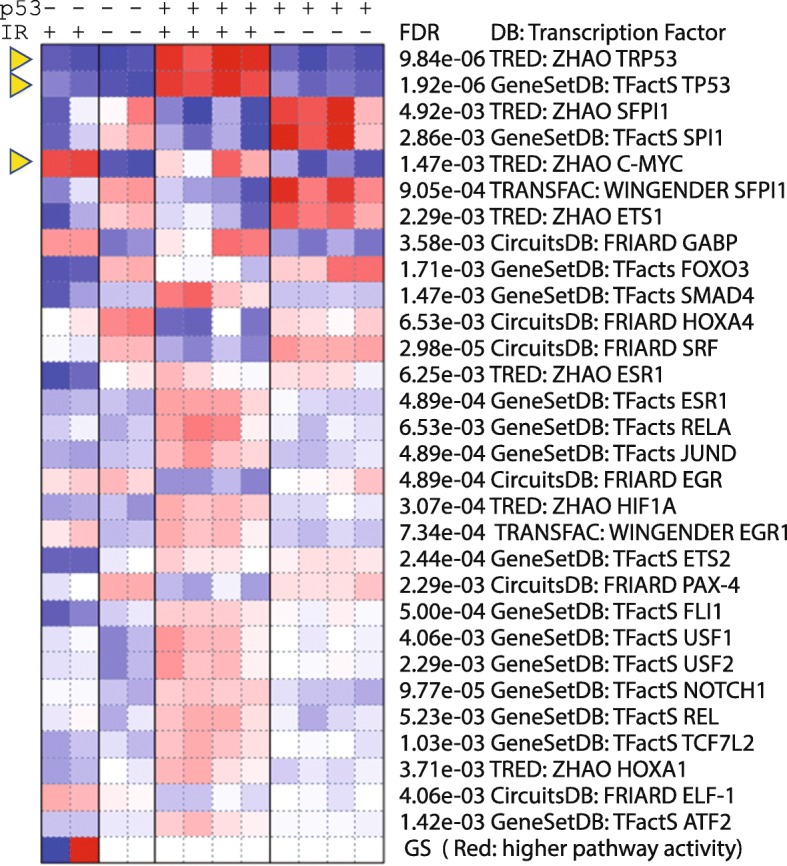
Differentially regulated TF target gene sets across sample types, identified by PGSEA

Many of the above observations can be confirmed by using pathway analysis based on the fold-change values of all genes. The results of GSEA on the interaction term can be found in Additional file 1: Table S15. The PGSEA package offers a convenient way to visualize the activities of pathways across all samples. Additional file 3: Figure S19 clearly shows that p53 signaling pathway, apoptosis, and positive regulation of cell cycle arrest are uniquely activated by IR in wild-type B cells. This is again confirmed by TF target genes (Fig. 15). In addition, the p53-independent upregulation of MYC target genes can also be observed in Fig. 15. Several ETS transcription factors, including SFPI1, SPI1, and ETS1, are suppressed by IR in both cell types. These factors may underlie the suppression of immune response as suggested [76]. Applying PGSEA on miRNA target genes highlights miRNA-30a (Additional file 3: Figure S20), whose target genes are specifically activated by IR in wild-type B cells. miRNA-30a was shown to be involved in response to IR [77] and mutually regulate p53 [78]. Thus, the complex p53 signaling pathways are unveiled with remarkable accuracy.

The upregulated p53 target genes can be seen in the KEGG pathway diagram (Additional file 3: Figure S21). This pathway map shows multifaceted roles of p53 in the regulation of apoptosis, cell cycle, DNA damage repair, and growth arrest. Many of these functions were re-discovered in our analyses above. This shows the power of comprehensive pathway databases coupled with broad analytic functionalities accessible via an intuitive user interface. Without iDEP, it can take days or weeks to write code and collect data to conduct all the analyses above. With iDEP, biologists can complete such analyses in as little as 20 min.

## Discussions

Taking advantage of the Shiny platform, we were able to pack many useful functionalities into iDEP, including high-quality graphics based on ggplot2 and interactive plots using Plotly. Compared with traditional web applications, Shiny has its drawbacks and limitations. The interface is not as flexible as those developed using JavaScript. Nevertheless, we believe an integrated web application like iDEP is a valuable tool to both bench scientists and bioinformaticians.

**Fig. 16:**
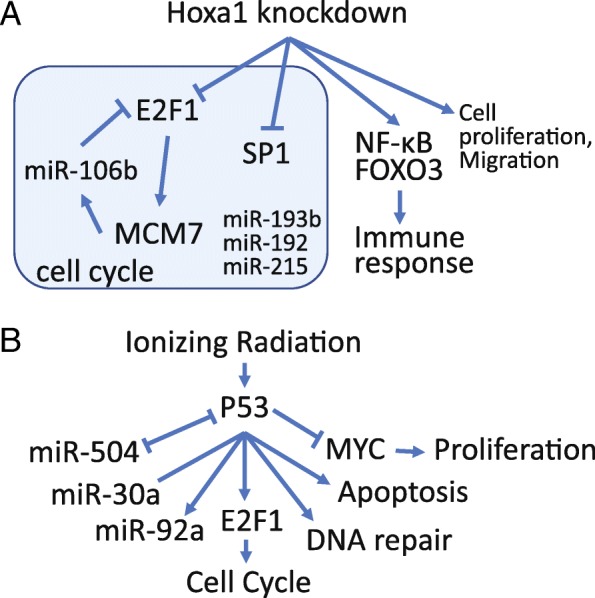
Bioinformatics analysis using iDEP generates many hypotheses regarding the molecular pathways underlying (**a**) Hoxa1 knockdown, and (**b**) Ionizing radiation

As an example, we extensively analyzed an RNA-Seq dataset involving Hoxa1 knockdown by siRNA in lung fibroblasts, and identified the down-regulation of cell-cycle genes, in agreement with previous analyses and experimental confirmation. Our analyses also show E2F and SP1 binding motifs are enriched in the promoters of downregulated genes, mediating the cell cycle arrest. Furthermore, we also find evidence that microRNAs (miR-17-5P, miR-20a, miR-106a, miR-192, miRNA-193b, and miR-215) might work with E2F factors to block the G_1_/S transition in response to reduced Hoxa1 expression. Interestingly, miR-106a is located in the intron of Mcm7, an E2F1 target gene. DEGs are also enriched with genes related to neuron parts, synapse, as well as neurodegenerative diseases. This is consistent with reports of Hoxa1’s role in neuron differentiation [36–38]. Hoxa1 knockdown induces expression of genes associated with the cytokine-cytokine interaction, lysosome, and cell migration, probably in response to the injected siRNAs. These genes are overrepresented with target genes of NF-κB, known to be involved in immune response. By combining both annotation dataset and analytic functionality, iDEP help biologists to quickly analyze their data to form new hypotheses (Fig. 16a).

In the second example, our analysis shows that in B cell without p53, radiation treatment upregulates MYC oncogenic pathway, triggering downstream genes with highly coherent functions such as cell proliferation, ribosome biogenesis, and ncRNA metabolism. Enriched with target genes of miR-124 and ETS domain transcription factors, genes downregulated by IR in p53 null B cells are associated with immune response, GTPase activity and actin cytoskeleton. In wildtype B cells, a p53-dependent transcriptional response to IR is evidently related to p53-mediated apoptosis and DNA repair, as expected. The target genes of MYC and E2F1 are suppressed by p53, leading to growth and cell cycle arrest (Fig. 16b). iDEP helps unveil the multifaceted functions of p53, and also highlight the potential involvement of several miRNAs (miR-92a, miR-504, and miR-30a).

Users should be cautious when interpreting results from pathway analysis, which can be obtained through the many combinations of methods and gene set databases. The biomedical literature is large and heterogeneous [79], making it easy to rationalize and make a story out of any gene. True pathways, like the effect of Hoxa1 knockdown on cell cycle, should be robustly identified across different methods and databases. Also, as demonstrated in the two examples, for each enrichment or pathway analysis, we tried to focus on the most significant gene sets.

## Conclusions

By integrating many Bioconductor packages with comprehensive annotation databases, iDEP enables users to conduct in-depth bioinformatics analysis of transcriptomic data through a GUI. The two use cases demonstrated that it can help pinpoint molecular pathways from large genomic datasets, thus eliminating some barriers for modern biologists.

Besides RNA-Seq and DNA microarray data, users can also use iDEP to analyze fold-change and FDR values calculated by other methods such as cuffdiff [80]. For unannotated genomes, iDEP can be used for EDA and differential expression analysis. For single-cell RNA-Seq data [81], only smaller, pre-processed datasets with hundreds of cells can be analyzed, as iDEP is mostly designed to handle transcriptomic data derived from bulk tissues.

In addition to updating the annotation database from Ensembl every year, we plan to continue to compile pathway databases for model organisms, similar to MSigDB and GSKB. For unsupported species, we will consider ways to incorporate user-submitted gene annotation. Based on user request and feedback, we will also add more functions by including additional Bioconductor packages.

## Methods

Figure 1 outlines the iDEP workflow. Expression matrix is first filtered, transformed and converted to Ensemble gene IDs, which are used internally to identify genes. The pre-processed data is then used for EDA, with methods such as K-means clustering, hierarchical clustering, principal component analysis (PCA), and t-SNE [32]. Gene clusters identified by K-means are analyzed by enrichment analysis based on a large gene annotation and pathway database. The identification of DEGs is done with either the *limma* [82] or DESeq2 [10] packages. This is also followed by enrichment analysis on the DEGs. The fold-change values are then used in pathway analysis using several methods.

To enable gene ID conversion, we downloaded all available gene ID mappings for 220 species from Ensembl [26, 27] (Additional file 1: Table S1), including 98 from Ensembl (vertebrates, release 91), 53 from Ensembl Plants (release 37) [28], and 69 from Ensembl Metazoa (release 37). The final mapping table for the current iDEP v0.72 release consists of 135,832,098 rows, mapping various gene IDs (Additional file 1: Table S2) into Ensembl. For example, 67 types of human gene IDs can be converted to Ensembl gene IDs. Besides common ID like gene symbol, Entrez, Refseq, UCSC, UniGene, and Interpro IDs, the 67 kinds of human gene IDs also include probe IDs for popular DNA microarray platforms, making it possible to re-analyze thousands of microarray datasets available at public repositories.

When multiple gene IDs are mapped to the same ENSEMBL gene, only the one with largest standard deviation is kept. Gene IDs not recognized by iDEP will be kept in the data using original gene IDs. Users can also avoid gene ID conversion by checking the “Do not convert gene IDs to Ensembl” checkbox in the “Pre-Process” page. This is useful when the user’s data is already Ensembl gene IDs, or the user just wants to conduct EDA and identify differentially expressed genes (DEGs).

In the pre-processing stage, gene IDs are first compared to all gene IDs in the database for 220 organisms. This enables automatic ID conversion and species identification. Genes expressed at very low levels are removed and data are transformed as needed using one of several methods. iDEP enforces log-transformation when a highly skewed distribution is detected. This type of mechanisms can help avoid issues in downstream analyses. The pre-processing stage also generates diagnostic and summary plots to guide users to make their choices.

EDA enables the users to explore variations and patterns in the dataset as a whole [83]. The main methods include hierarchical clustering with heatmap, k-means clustering, and PCA. Enrichment analysis of genes derived from k-means clustering is conducted to gain insights into the functions of co-expressed genes. Initial attempts of pathway analysis are carried out using the PCA loadings on each gene. This can tell us the biological processes underlying each direction of expression change defined by the principal components.

Differential expression analysis relies on two Bioconductor packages, *limma* [82] and DESeq2 [10]. These packages can meet the needs for most studies, including those involving multiple biological samples and factorial design. See [84] for detailed review of other methods and consideration of sample size and variance. Normalized expression data is analyzed using *limma*. Read counts data can be analyzed using three methods, namely limma-trend [14], limma-voom [14, 85], and DESeq2. Other methods such as edgeR [13] may be incorporated in the future.

For simple study designs, iDEP runs differential gene expression analysis on all pairs of sample groups, which are defined by parsing sample names. For complex studies, users can upload a file with experiment design information and then build statistical models that can involve up to 6 factors. This also enables users to control for batch effects or dealing with paired samples.

Fold-change values for all genes returned by *limma* or DESeq2 are used in pathway analysis using GSEA [86], PAGE [33, 34], GAGE [57] or ReactomePA [87]. Taking advantage of centralized annotation databases for 98 species at Ensembl (release 92), 53 in Ensembl Plants (release 40), and 69 in Ensembl Metazoa (release 40), we downloaded not only GO functional categorizations, but also promoter sequences for defining transcription factor (TF) binding motifs for most species. Metabolic pathways were downloaded directly from KEGG [21] for 131 species (Additional file 1: Table S1). Also, we incorporated Pathview package [58] to show gene expression on KEGG pathway diagrams downloaded via API. In addition, we also included many species-specific pathway knowledgebases, such as Reactome [87, 88], GeneSetDB [89] and MSigDB [39] for human, GSKB for mouse [29], and araPath for Arabidopsis [53]. These databases contain diverse types of gene sets, ranging from TF and microRNA target genes, protein-protein interactions, to manually curated lists of published DEGs. For the human genome, we collected 140, 438 gene sets (Table 2). Such large, diverse databases enable in-depth analysis of expression data from different perspectives. Table 2 contains databases that we deemed useful. For human pathways, many other databases and tools exists [90–92].

The PGSEA package [33] implements the Parametric Analysis of Gene Set Enrichment (PAGE) algorithm [34] to display the activities of pathways in individual samples in terms of Z scores, which characterize how much the mean of the fold-changes for genes in a certain pathway deviates from the mean observed in all the genes. We modified the PGSEA code by adding an analysis of variance (ANOVA) on the Z scores across sample groups. Also, after cutoff with FDR, pathways are ranked by the standard deviation. This modification yields meaningful, intuitive display of differentially regulated pathways across sample groups.

PCA enables us to project samples into two-dimensional space. We also treated the PCA loadings onto each of the genes as expression data to run pathway analysis with the PGSEA package. For each pathway, this runs the PAGE algorithm which performs one-sample t-test on each gene set.The adjusted *P*-values are used to rank the pathways for each of the first 5 principal components. The pathways are labeled with FDR first, followed by the principal components (PC1, PC2 and so on). Only 5 pathways for each principal component are shown, but duplicated ones are skipped.

iDEP also enables users to retrieve protein-protein interaction (PPI) networks among top DEGs via an API access to STRING [24]. These networks can be rendered both as static images and as richly annotated, interactive graphs on the STRING website. The API access also provides enrichment analysis (GO, KEGG, and protein domains) for 115 archaeal, 1678 bacterial, and 238 eukaryotic species, thus greatly expanding the species coverage of iDEP.

Based on their chromosomal location obtained from Ensembl, we visualize fold-changes of genes on all the chromosomes as an interactive graph based on Plotly. iDEP can also use the PREDA package [62] to detect chromosomal regions overrepresented with up- or down-regulated genes. This is useful for studies such as cancer that might involve chromosomal deletion or amplification.

For larger datasets, users can use bi-clustering algorithms to identify genes with correlated expression among a subset of samples, using the 8 methods implemented in 3 Bioconductor packages biclust [93], QUBIC [94], and runibic [95]. Gene co-expression networks can also be constructed with the WGCNA package [96]. Enrichment analysis is routinely conducted on gene clusters derived from these methods.

To identify enriched TF binding motifs, transcript annotation and promoter sequences are retrieved from Ensembl. For genes with multiple transcripts, the transcription start site (TSS) with multiple transcripts is used. If multiple TSS locations have the same number of transcripts, then the most upstream TSS is used. Promoters are pre-scanned using TF binding motifs in CIS-BP [52]. Instead of defining a binary outcome of binding or not binding, which depends on arbitrary cutoffs, we recorded the best score for each of the TFs in every promoter sequence. Then student’s t-test is used to compare the scores observed in a group of genes against the rest of genes. The P-values are corrected for multiple testing using false discovery rate (FDR).

To enhance reproducibility in research, we will make older versions of iDEP software and database for each significant upgrade. iDEP also produces an R and R Markdown file which captures users’ parameterization during the analysis. These files could be downloaded, alongside related database files, to reproduce their analysis.

The Shiny package by RStudio provides a powerful web framework for developing applications using R. We used docker containers to configure and manage the Shiny server. Containerization also enables us to easily deploy the service and scale up to take advantage of multiple cores. Load balanced with Nginx, our web server can handle hundreds of concurrent users by distributing jobs to dozens of R processes. The source code for iDEP and our server configuration files are available at our GitHub repository [97]. Detailed documentation of iDEP, including video tutorial and a full list of supported species, is available at [98].

## Additional files

Additional file 1: **Tables S1-S16.** **Table S1.** contains list of 220 species covered by current version of iDEP. **Table S2.** include the 2196 types of gene IDs that can be recognized. **Tables S3-S16.** are results from the analyses of two example datasets. (XLSX 1970 kb)

Additional file 2: Read count file for Hoxd1 knockdown example. This file is derived from short read archive (SRA) SRP012607 using Sailfish. (CSV 718 kb)

Additional file 3: **Figures S1-S21**. Results from the two example datasets. (PDF 2973 kb)

Additional file 4: DNA microarray data of thymic tissue of down syndrome infants. Data is from GSE69210 from NCBI. (CSV 4745 kb)

Additional file 5: An example of customized R code generated by iDEP. This code is generated for the analysis of the Hoxa1 dataset. (R 11 kb)

Additional file 6: An example of R Markdown file generated by iDEP. This code is generated for the analysis of the Hoxa1 dataset. (RMD 15 kb)

Additional file 7: Core R functions in iDEP. This code is generated for the analysis of the Hoxa1 dataset. (R 172 kb)

Additional file 8: Read count file for the mouse ionization/p53 dataset. This file was used in our analysis. (CSV 1513 kb)

Additional file 9: Experiment design file for the mouse ionization/p53 dataset. This file was used in our analysis. (CSV 226 bytes)

## Abbreviations

CCRI: Cytokine-cytokine receptor interaction
DEG: Differentially expressed gene
EDA: Exploratory data analysis
FC: Fold-change
FDR: False discovery rate
FPKM: Fragments Per Kilobase Million
GAGE: Generally Applicable Gene-set Enrichment
GGO: Gene Ontology
iDEP: Integrated Differential Expression and Pathway analysis
IR: Ionizing radiation
MDS: multidimensional scaling
PAGE: Parametric Analysis of Gene Set Enrichment
PCA: Principal component analysis
Rlog: Regularized log
RNA-seq: RNA sequencing
siRNA: Small interfering RNA
START App: Shiny Transcriptome Analysis Resource Tool
TSS: Transcription start site
