# Supplementary material for: iDEP: an integrated web application for differential expression and pathway analysis of RNA-Seq data
Source: BMC Bioinformatics. 2018 Dec 19;19:534. doi: 10.1186/s12859-018-2486-6 (PMC6299935; doi:10.1186/s12859-018-2486-6)
Supplement: Supplementary file 3 — Figures S1-S21. Results from the two example datasets. (PDF 2973 kb) [file 12859_2018_2486_MOESM3_ESM.pdf]

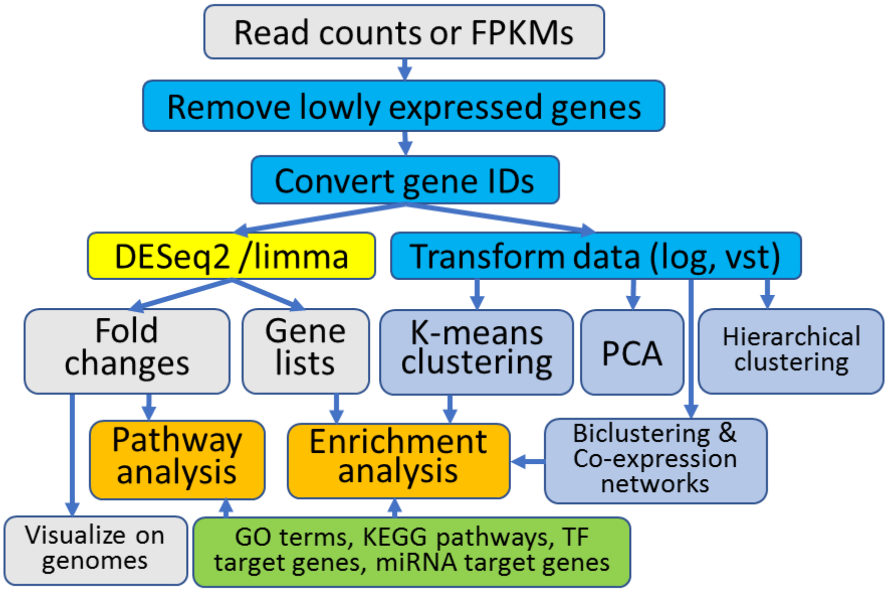

Supp. Figure 1. A flowchart for the analysis of two example datasets.

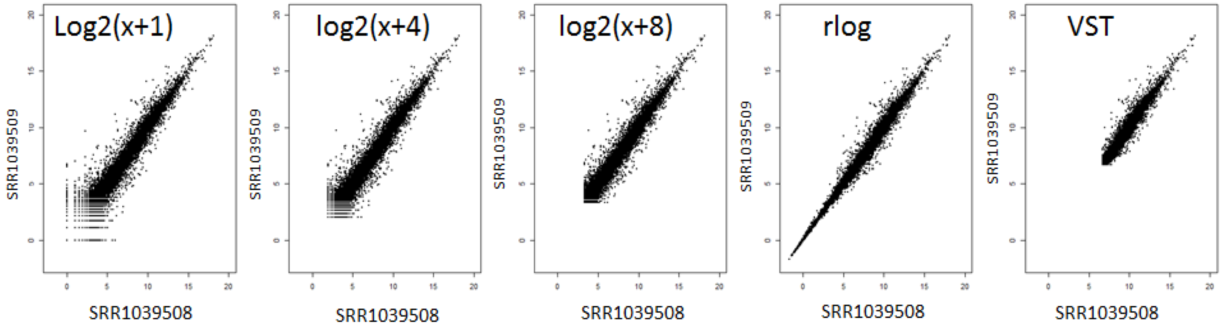

Supp. Figure 2. Effect of various data transformations on RNA-Seq read count data. The two libraries are from different biological samples. Transformations drastically alter lowly expressed genes.

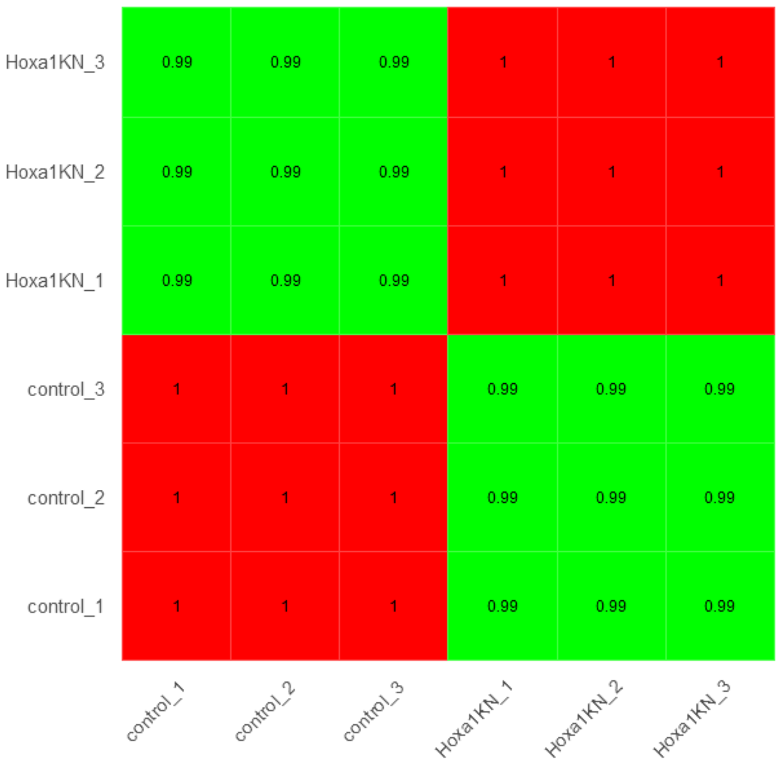

Supp. Figure 3. Correlation matrix of the Hoxa1 knockdown dataset. The numbers are Pearson's correlation coefficients.

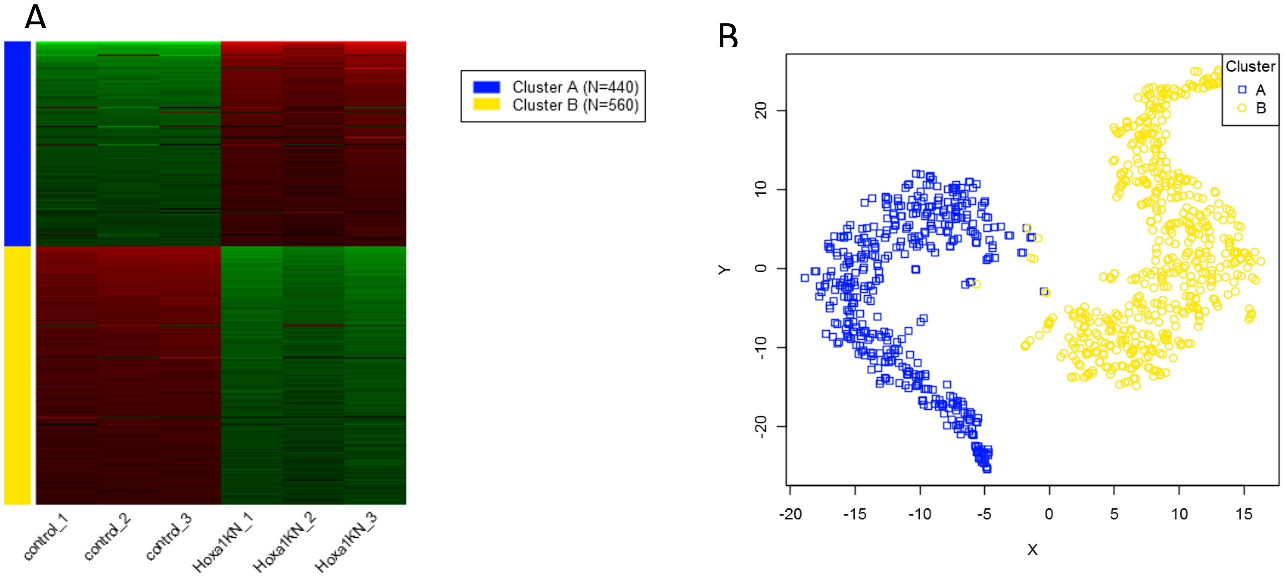

Supp. Figure 4. Results of k-means clustering (A), and a t-SNE mapping (B) of the top 1000 genes in the Hoxa1 knockdown dataset.

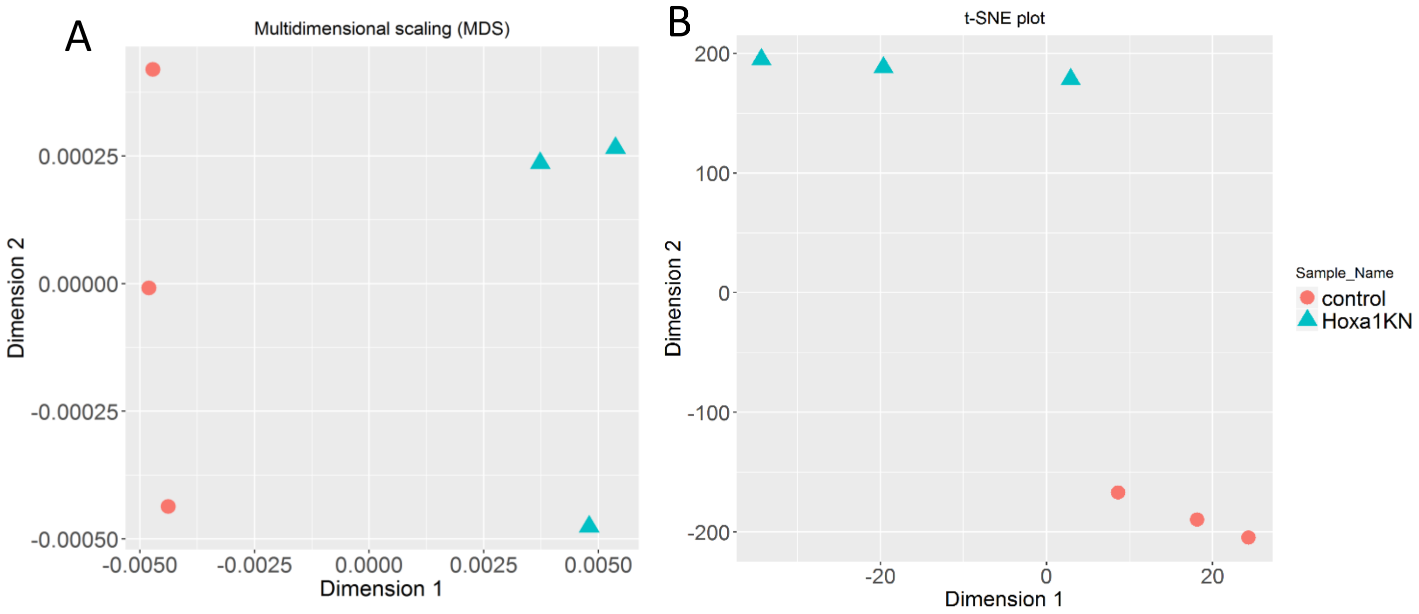

Supp. Figure 5. Mapping samples using (A) MDS and (B) t-SNE in the Hoxa1 knockdown dataset.

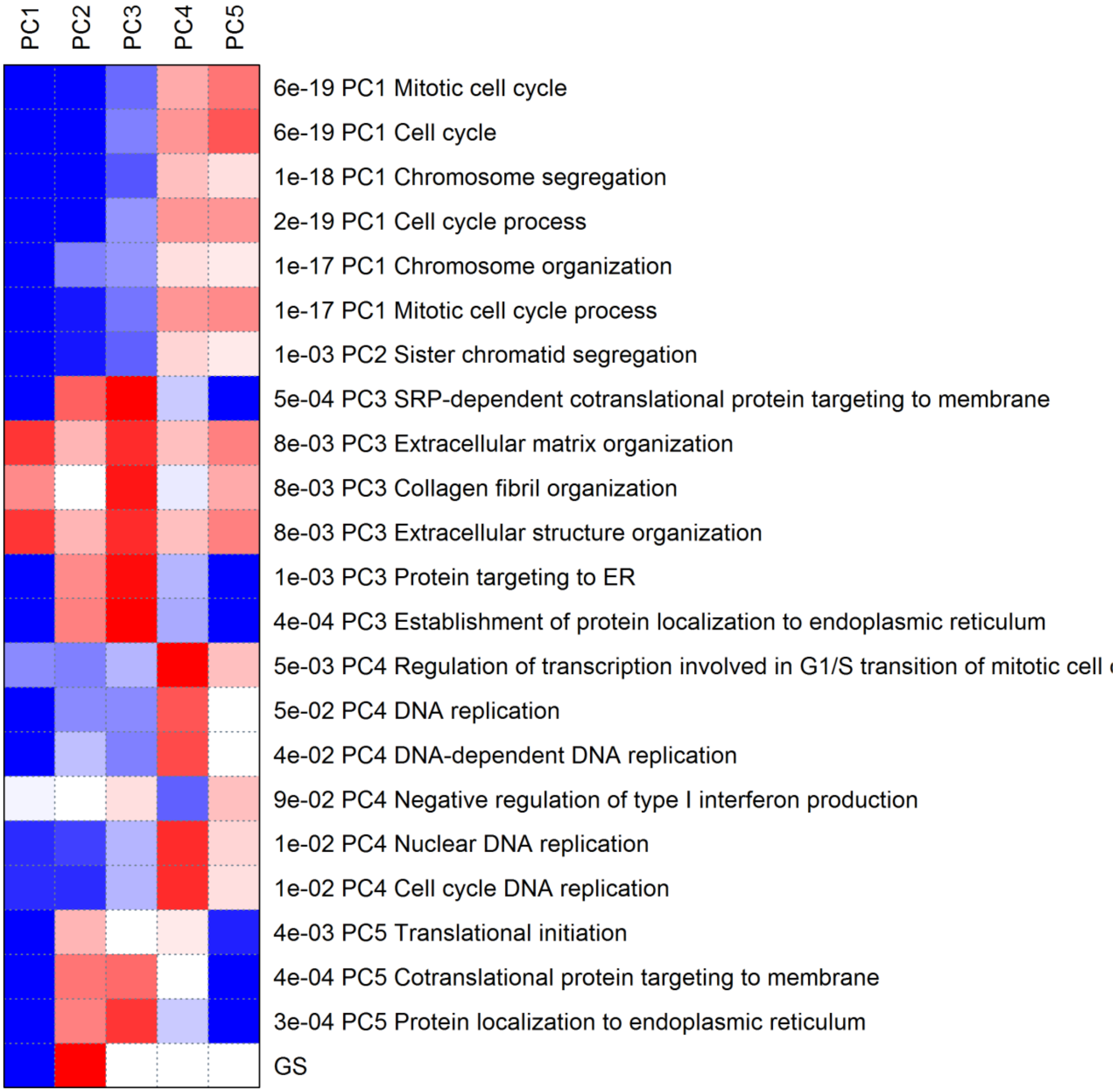

Supp. Figure 6. Pathway analysis using PGSEA on PCA components in the Hoxa1 knockdown dataset.

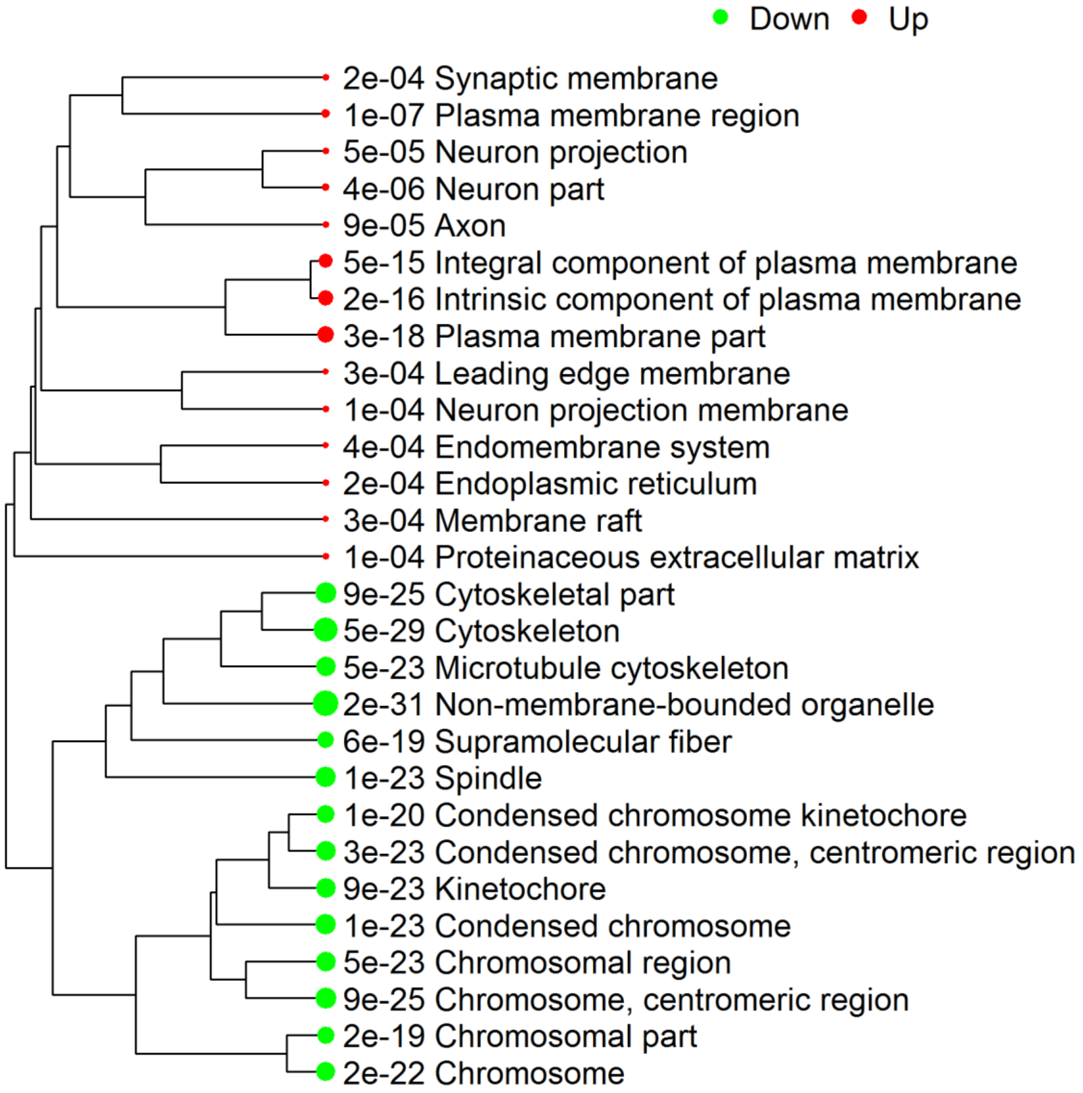

Supp. Figure 7. Enriched GO Cellular Component terms in DEGs of the Hoxa1 knockdown dataset.

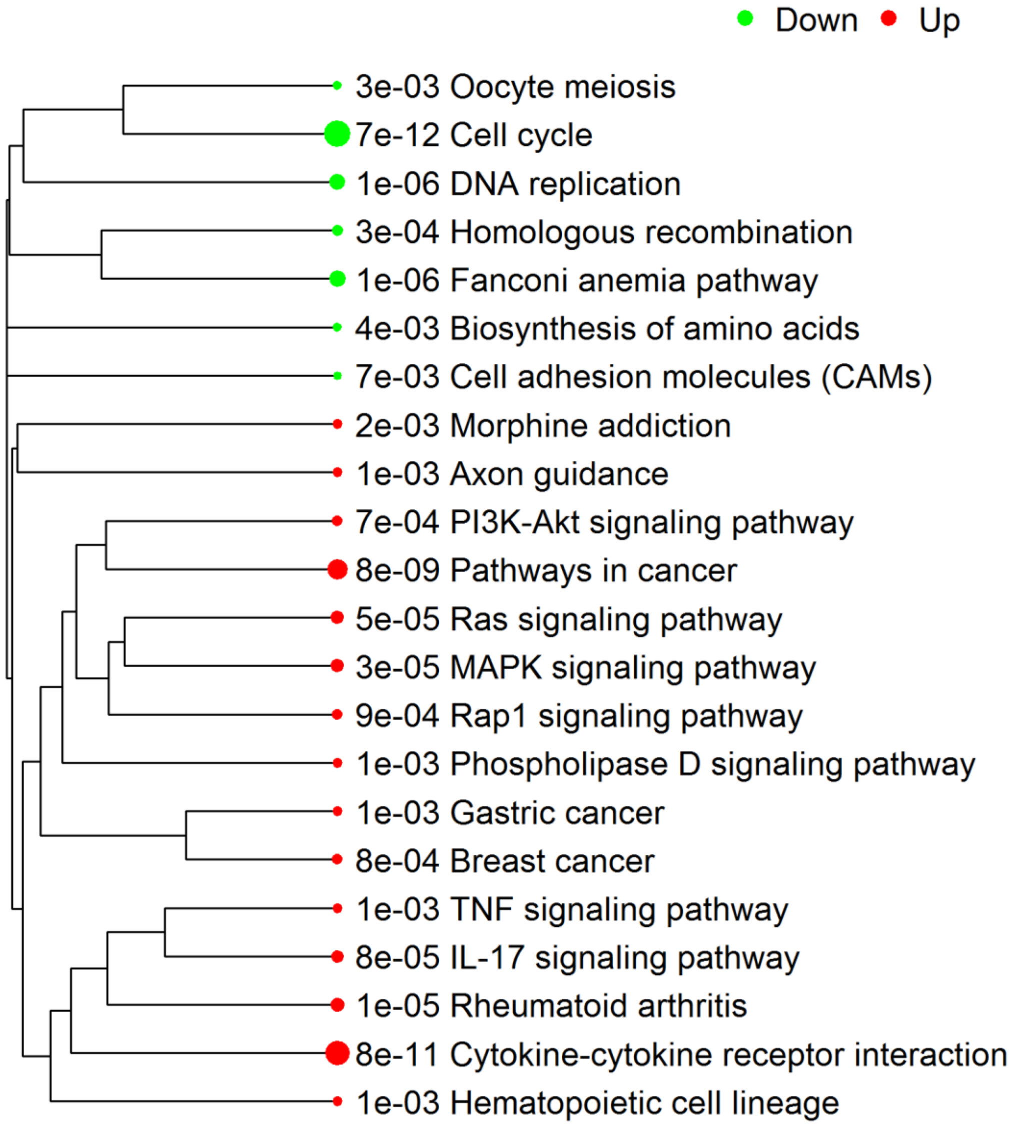

Supp. Figure 8. Enriched KEGG pathways in DEGs in the Hoxa1 knockdown dataset.

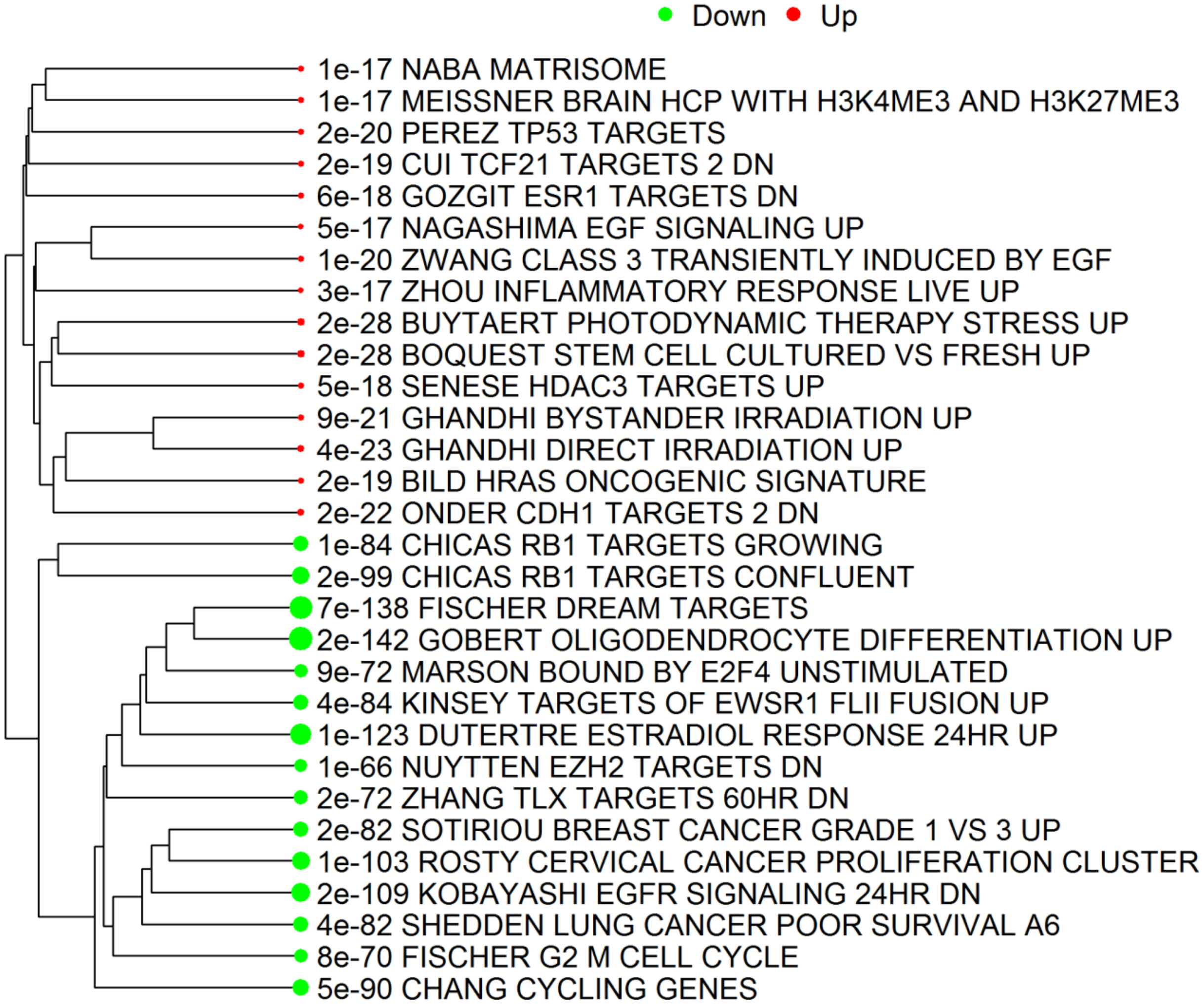

Supp. Figure 9. Overlaps of DEGs in the Hoxa1 knockdown dataset with MSigDB curated pathways, including published gene expression signatures.

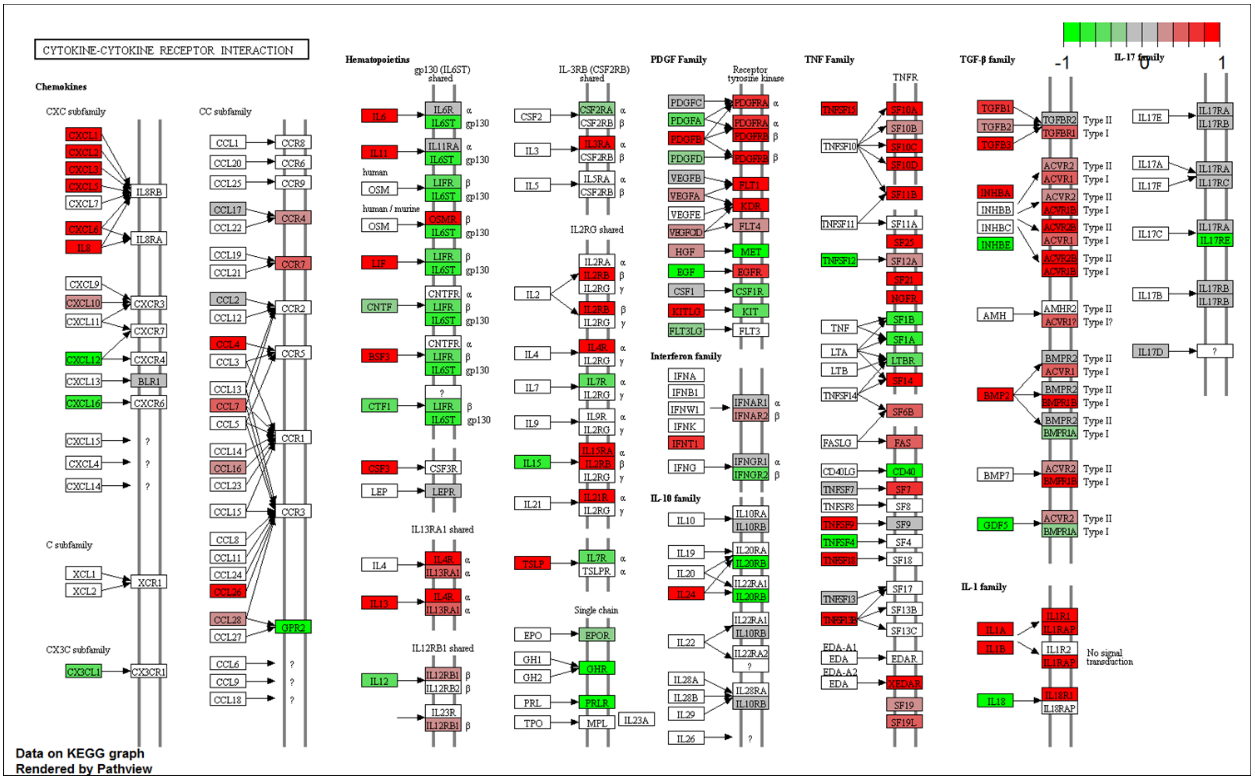

Supp. Figure 10. Genes involved in Cytokine-cytokine receptor interaction are mostly up-regulated (red) when Hoxa1 is knocked down. Fold-change information is color-coded on KEGG pathway diagram.

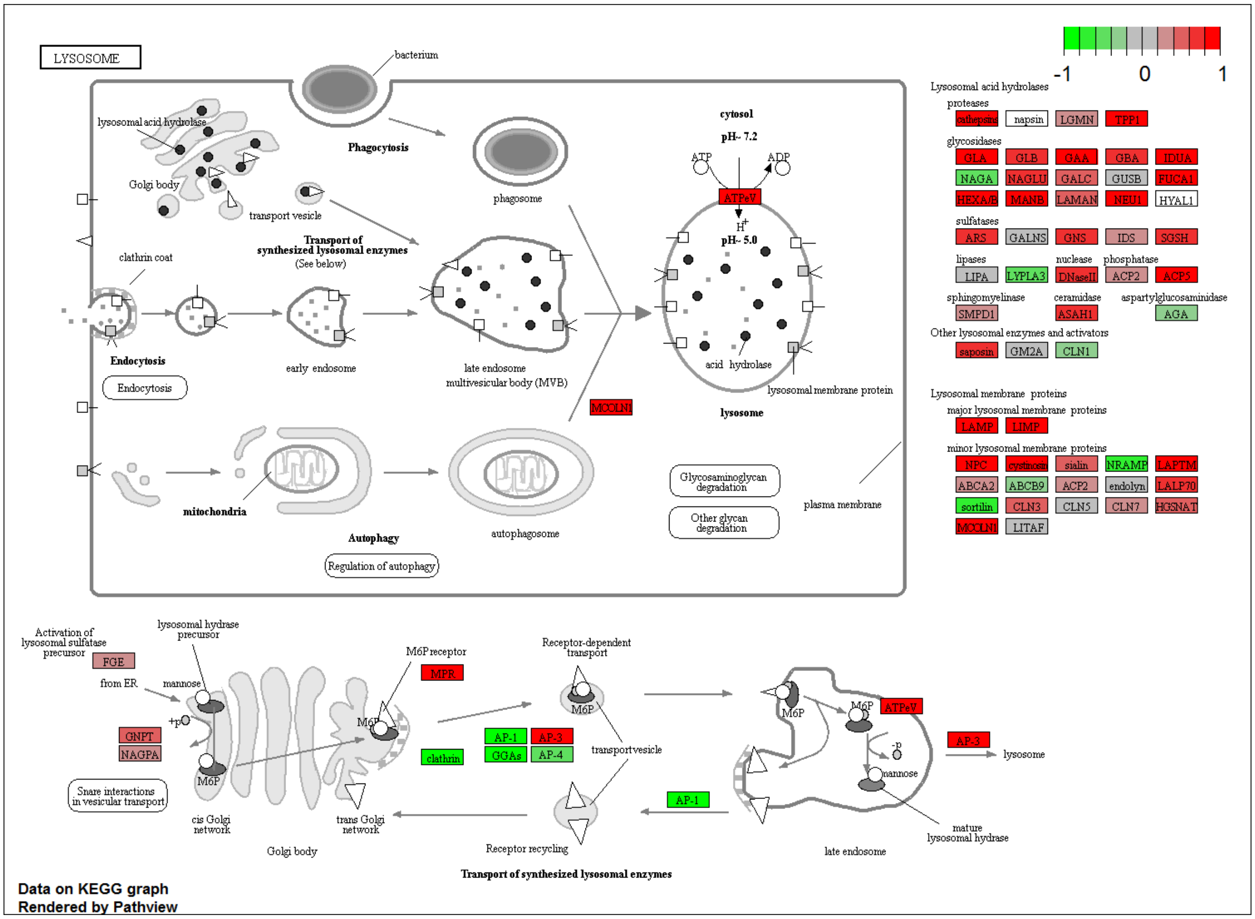

Supp. Figure 11. Lysosome related genes are upregulated (red) by Hoxa1 knockdown.

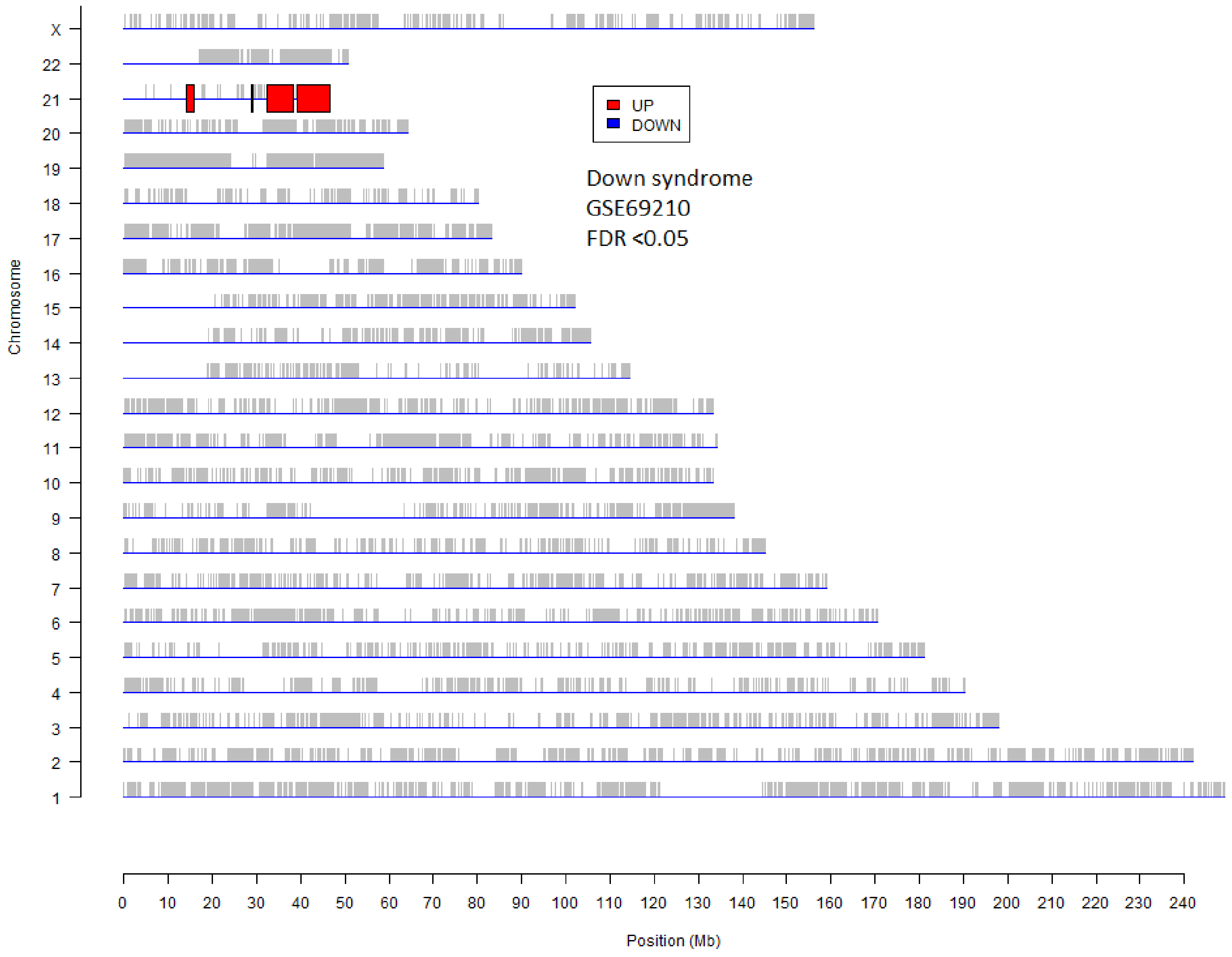

Supp. Figure 12. PREDA detects upregulated chromosomal regions on Chr. 21 in samples with down syndrome.

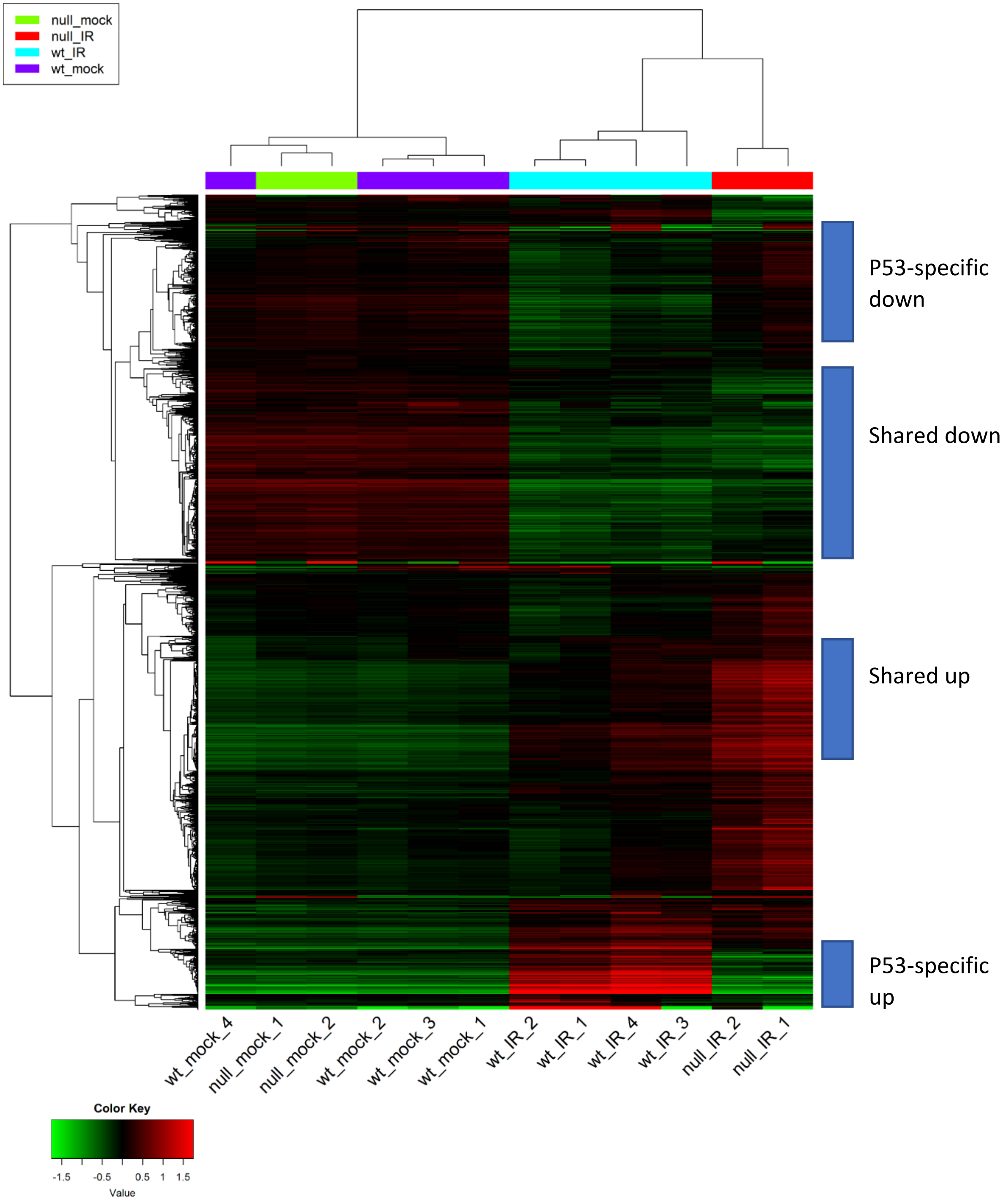

Supp. Figure 13. Hierarchical clustering of the 2500 genes shows the patterns of various groups of genes.

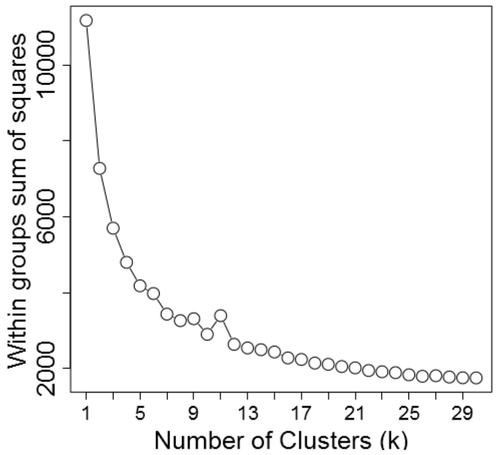

Supp. Figure 14. Plot of within groups sum of squares for the determination of number of clusters. According to the elbow method, one should choose a k such that additional clusters does not substantially reduce sum of squares. This can lead to a k of 5. But we found it is helpful to use k that is slightly larger than this. K=9 is chosen.

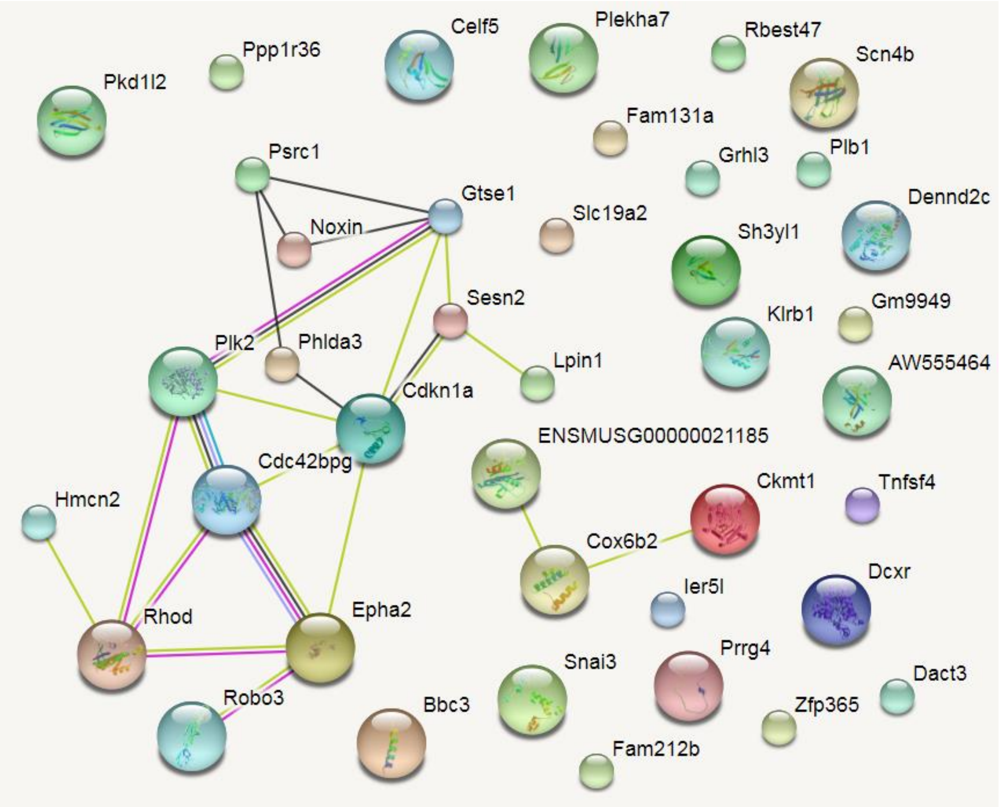

Supp. Figure 15. Protein-protein interaction (PPI) network among the top 40 genes upregulated by IR in p53 wildtype B cells.

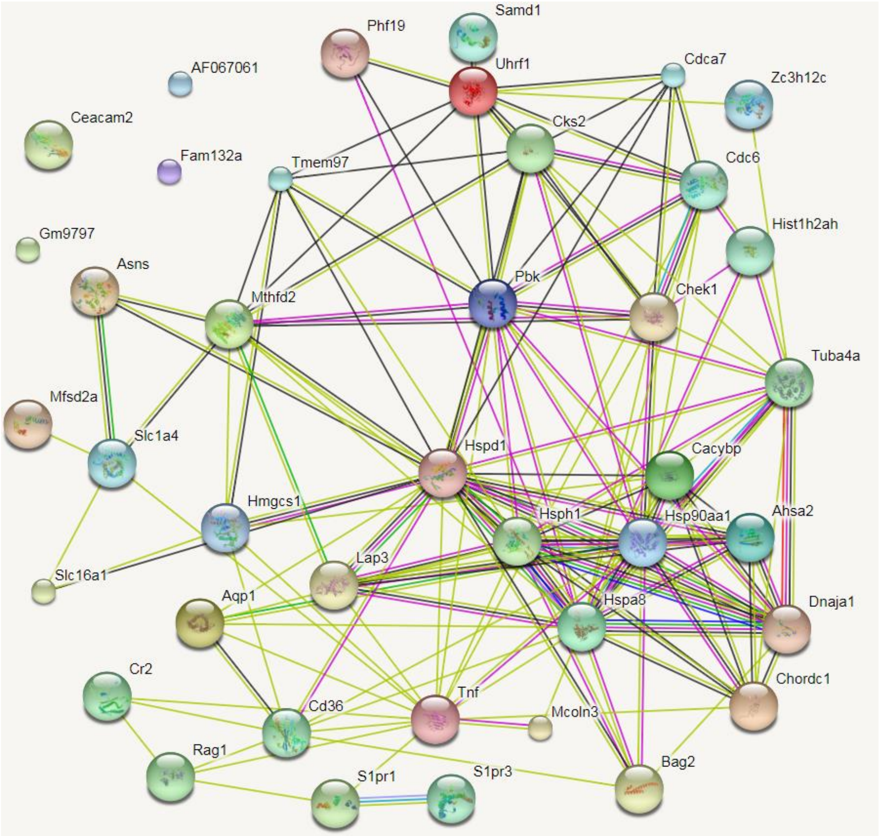

Supp. Figure 16. PPI network of the top 40 downregulated genes in wildtype B cells when treated with IR. These are more densely connected.

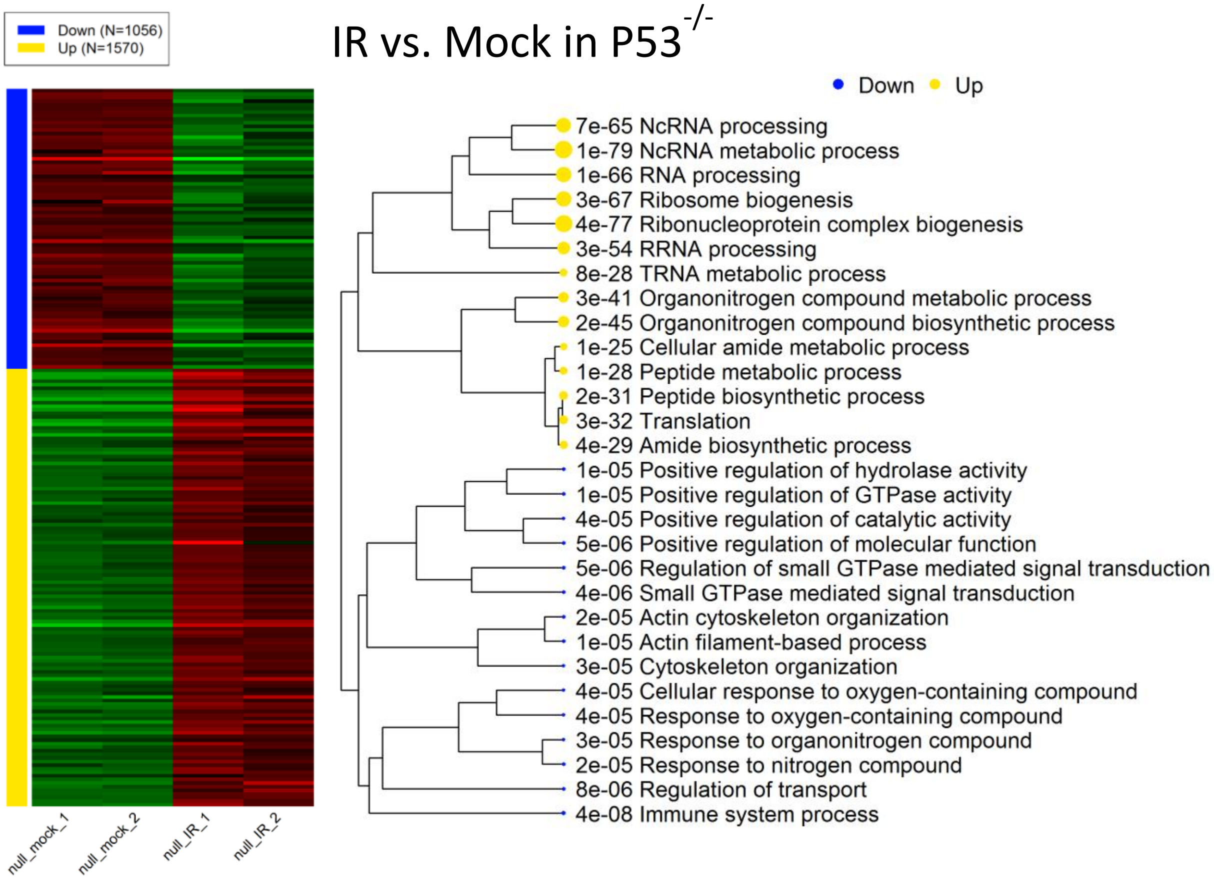

Supp. Figure 17. Effect of IR on p53 null samples. A) The expression patterns of DEGs. B) Up-regulated genes are highly overrepresented with ribosome biogenesis, RNA, especially ncRNA, processing, and translation. Downregulated genes are enriched genes involved with immune system, actin cytoskeleton, and GTPase activity.

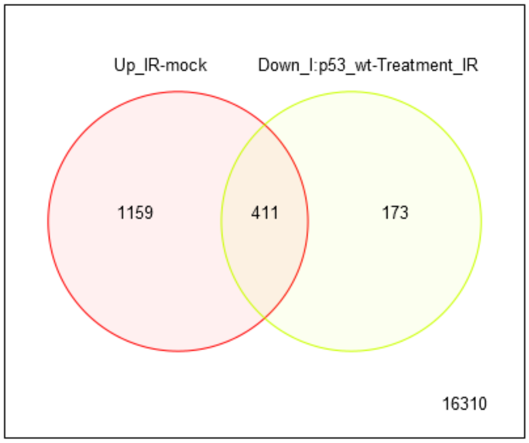

Supp. Figure 18. Genes downregulated in the interaction term is mostly included in those upregulated by IR in p53 null B cells.

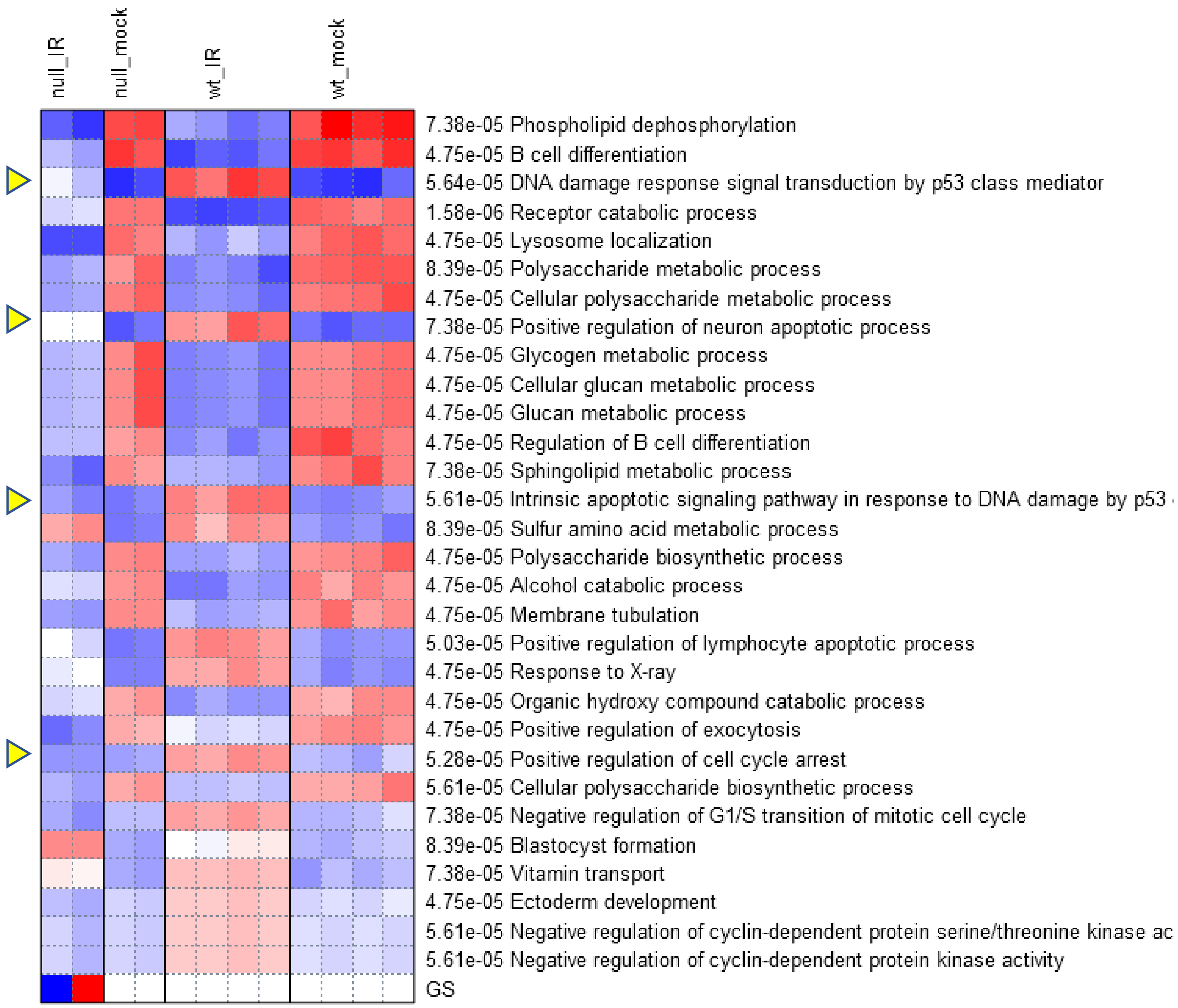

Supp. Figure 19. PGSEA visualization of pathway activities using GO biological processes.

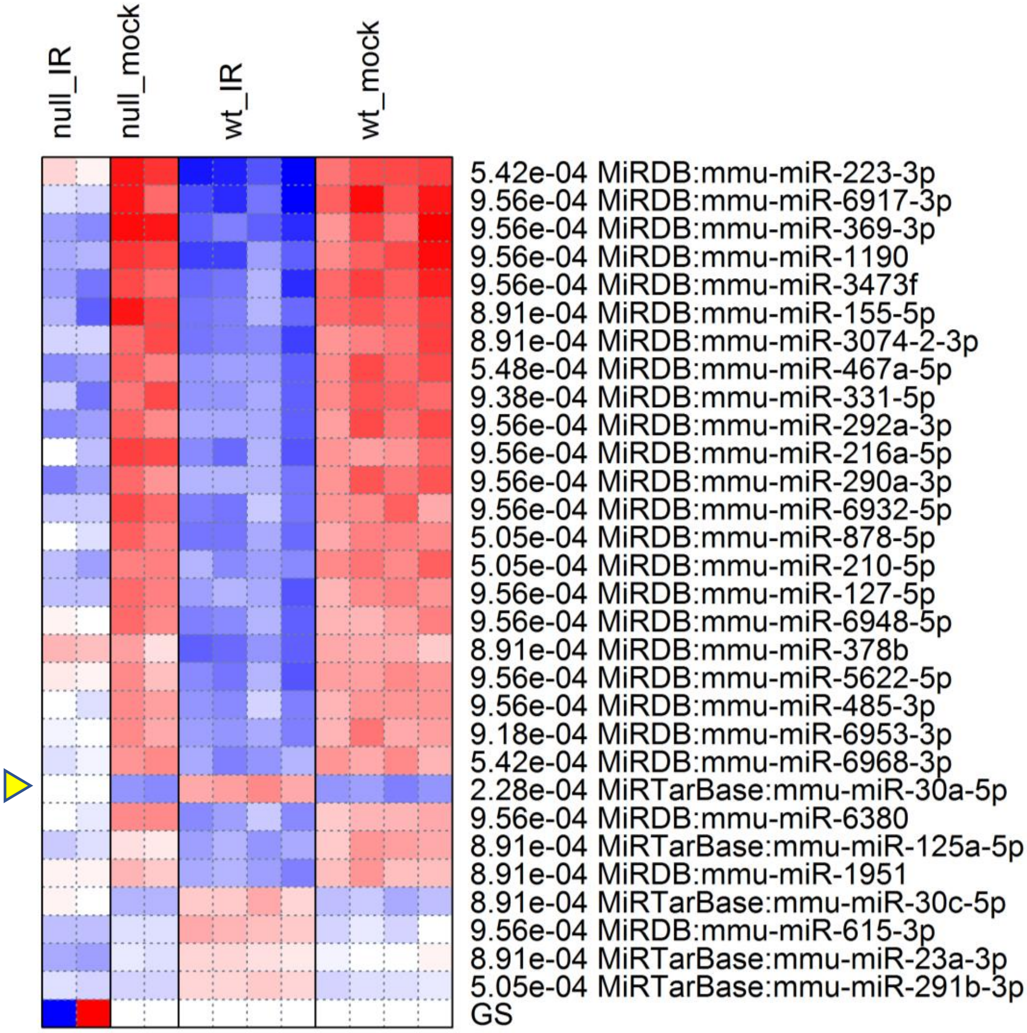

Supp. Figure 20. Average expression levels of miRNA target genes using PGSEA package.

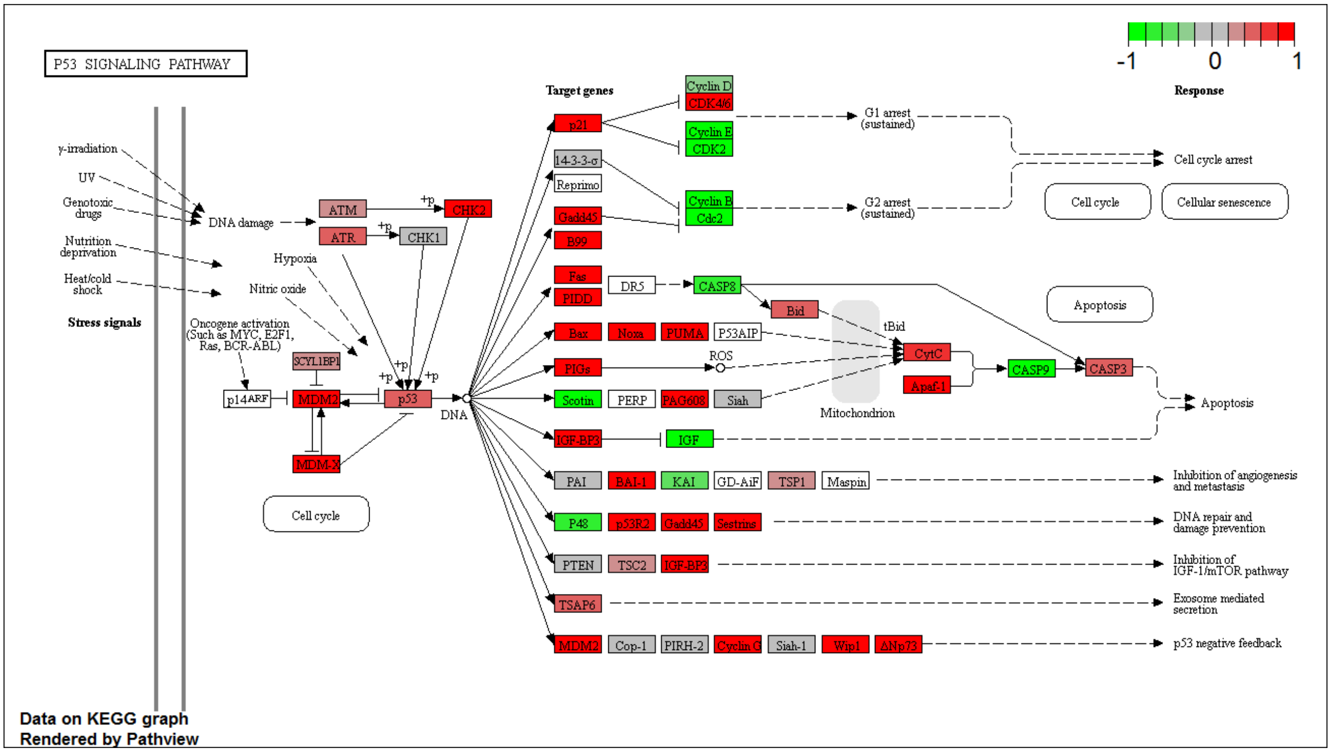

Supp. Figure 21. Fold changes of genes in P53 signaling pathway in wildtype B cells treated with IR. Upregulation (red) of many key-players.
